# Computational Analysis of Rho GTPase Cycling

**DOI:** 10.1371/journal.pcbi.1002831

**Published:** 2013-01-10

**Authors:** Cibele Vieira Falkenberg, Leslie M. Loew

**Affiliations:** Center for Cell Analysis and Modeling, University of Connecticut Health Center, Farmington, Connecticut, United States of America; North Carolina State University, United States of America

## Abstract

The Rho family of GTPases control actin organization during diverse cellular responses (migration, cytokinesis and endocytosis). Although the primary members of this family (RhoA, Rac and Cdc42) have different downstream effects on actin remodeling, the basic mechanism involves targeting to the plasma membrane and activation by GTP binding. Our hypothesis is that the details of GTPase cycling between membrane and cytosol are key to the differential upstream regulation of these biochemical switches. Accordingly, we developed a modeling framework to analyze experimental data for these systems. This analysis can reveal details of GDI-mediated cycling and help distinguish between GDI-dependent and -independent mechanisms, including vesicle trafficking and direct association-dissociation of GTPase with membrane molecules. Analysis of experimental data for Rac membrane cycling reveals that the lower apparent affinity of GDI for RacGTP compared to RacGDP can be fully explained by the faster dissociation of the latter from the membrane. Non-dimensional steady-state solutions for membrane fraction of GTPase are presented in multidimensional charts. This methodology is then used to analyze glucose stimulated Rac cycling in pancreatic β-cells. The charts are used to illustrate the effects of GEFs/GAPs and regulated affinities between GTPases and membrane and/or GDI on the amount of membrane bound GTPase. In a similar fashion, the charts can be used as a guide in assessing how targeted modifications may compensate for altered GTPase-GDI balance in disease scenarios.

## Author Summary

Among the functions of the small GTPases Rac, RhoA and Cdc42 are the regulation of protein traffic, insulin secretion, cell shape, survival and motility. The last two are important steps for tumor growth and metastasis. The function of these proteins relies on their expression levels, proper membrane localization and activation. In addition, all three proteins compete for the same protein ‘GDI’, which modulates their cycling. These proteins are ubiquitous in mammalian cells, but also studied in simpler *in vitro* systems and cultured yeast. Here we show, using a series of computational analyses, that for each of these experimental systems the dominant pathway for membrane cycling of GTPases seems to differ. This means that the researcher interested in the physiological function of any of those proteins must make sure that the experimental system is appropriate. We present a methodology to identify the dominant pathways by measuring the apparent membrane dissociation rate of the protein as a function of GDI concentration. We provide charts generated from parametric scans. This analysis is then applied to the Rac-dependent insulin secretion pathway in pancreatic ß-cells, revealing that direct signaling between Rac and the membrane is an essential mechanism that emerges from the data.

## Introduction

The activity of small GTPases RhoA, Cdc42 and Rac1 are controlled by spatial localization, nucleotide binding, and binding to Rho guanine nucleotide dissociation inhibitor (GDI). The importance of these three GTPases for cytoskeleton organization, cell migration and polarization is well established [1]–[3] and up/down regulation of GDI has been linked to metastatic and chemoresistant cancers [4], [5]. The spatial localization of these three GTPases is important for activation by membrane bound guanine nucleotide exchange factors (GEF), which promote GTPase release of GDP and binding to GTP; the GTP state activates and/or recruits effectors at the membrane, producing the downstream actin-mediated cellular response. The GTPase activating proteins (GAP) bind to the active GTPases and promote conversion of the nucleotide GTP into GDP, inactivating the GTPase. Binding to GDI promotes relocation of the GTPases from the membrane to the cytosol, inhibits interaction with effectors and inhibits exchange between GDP and GTP bound states (for more detailed review see [6]). In other words, the ratio between GEF/GAP activities determines the fraction of membrane bound GTPases that is available to interact with the effectors, while the interaction with GDI regulates the amount of GTPases available for activation. Another potential function of GDI is to protect the cytosolic fraction of GTPase from degradation [7].

The interactions between GDI's and GTPases can be regulated, modulating the cycling for spatial and temporal localization. For example, the affinities between GDI and GTPases may depend on nucleotide state [8], experimental conditions [9], post-translational modifications [10], phosphorylation state of GTPases and GDI [11], [12], sometimes resulting in translocation of the inactive GDI (that cannot bind GTPase) to the membrane [11]. The dependence of nucleotide state on GTPase membrane affinity has been studied via constructs mimicking its constitutively active and inactive forms. In yeast, it has been proposed that the cycling between active and inactive form also impacts the solubility of Cdc42 [13]. However, the constitutively active mutant Q61L used in this study seems to very poorly interact with GDI *in vivo* in comparison to *in vitro* [14]–[16]. In addition, the lipid composition of membranes can shift the GTPase population from cytosolic GDI-bound to membrane bound [17], [18].

As a further complication, the delivery and removal of GTPases from the membrane may also be independent of GDI. Mutants of Rac and Cdc42 that are unable to bind to GDI successfully promote membrane ruffling and actin reorganization in mammalian cells [19], [20]. Studies in polarized yeast indicate vesicular trafficking as an rdi1 (the GDI in yeast) independent mechanism for delivery of GTPase to the plasma membrane [21]. However, numerical analysis revealed that vesicular traffic alone will only result in GTPase polarization if there is a yet unknown mechanism for Cdc42 concentration in the trafficking vesicles [22].

A second GDI independent mechanism was revealed by *in vitro* experiments from Cerione's group. Cdc42 dissociates from the membrane at the same rate, whether in presence or absence of GDI; about 10% of RacGDP is translocated from the lipid membranes to the soluble fraction in absence of GDI, in contrast to negligible amounts for RacGTP or Cdc42 [23]. Similar results for Rac were obtained in control experiments against different GDI constructs and Sf9 cell membranes [11]. *In vivo* experiments on cultured fibroblasts also highlight that the removal rate of Rac from the membrane in those cells is not dependent on GDI. The Rac apparent membrane dissociation rate: a) is independent of GDI expression levels; b) is independent of spatial localization (protusive vs. quiescent regions); c) is dependent on nucleotide state; d) the reduced dissociation rate for active Rac is not due to elevated signaling [15]. In contrast, in cultured yeast, rdi1 promotes the fastest mechanism of Cdc42 membrane removal [21].

In order to integrate and reconcile these different observations, in this work we computationally analyze the mechanisms by which GTPases can be removed from and delivered to the membrane, estimating their relative contributions. To achieve this we initially developed a ‘lumped’ model that allows us to readily compare GDI-mediated and GDI-independent GTPase cycling. Simulation results over a wide range of parameters are conveniently displayed as contour plots. These charts serve as a visual tool to evaluate the effect of modifying the affinities between GTPases and membrane or GDI on the GTPase membrane bound fraction. The parameters of the lumped model reveal the function of GDI in GTPase cycling. We also develop an analysis of the role of vesicle trafficking in the mechanism for GTPase cycling in yeast. We then present a more detailed model for GTPase membrane cycling, explicitly accounting for the nucleotide state and interaction with effector proteins. This model permits us to derive all the rate constants involved in Rac cycling, from experimental data on cultured fibroblasts [15]. The functional form and parameters extracted for Rac cycling are consistent with the role of crosstalk, as emphasized by Burridge and colleagues [7]. In order to illustrate the importance of identifying such parameters in vivo, we analyze Rac membrane translocation in glucose stimulated β-cells. The analysis suggests that 2 mechanisms must be contributing to the delivery of Rac to the membrane: phosphorylation-mediated downregulation of the affinity between Rac and GDI and an increase in affinity between Rac and the membrane, possibly via lipid signaling [24]. Overall, our analysis demonstrates that the Rho-GTPase cycling can access qualitatively diverse pathways in different cellular systems or through different experimental manipulations.

## Methods

The computational methods used are extensively described in the supporting material Text S1. In summary, the detailed model was coded using BioNetGen [25], [26]. It was exported into Matlab (The MathWorks, Natick, MA) where the parametric search was performed using the function ‘fmincon’. Details on the initial guess, range of parameters searched, constraints, and criteria for optimization are described in Text S1. The compact model and steady state solutions for the example of Rac in β-cells where generated using Mathematica [27]. The dynamic model was originally created in BioNetGen and exported into Virtual Cell [28]–[30].

## Results

The overarching theme of the results we describe below is that the canonical Rho GTPase signaling relay can operate differently in different experimental and/or biological contexts. We divided our analysis of Rho GTPase cycling into subsections that describe individual mechanisms and then how they may be integrated. The common methodology used in each of the first three sections is the identification of non-dimensional groups and characterization of the system based on such variables. This methodology leads to a collective set of results correlating the parameters and variables of interest and identify regimes where a reduced system is valid. Sections Detailed Model and Example respectively apply these analyses to understand published data on Rac cycling and to make predictions on Rac cycling in an unexplored cellular system. The variables and parameters used throughout the text are summarized in Tables 1–3.

**Table 1:** Variables and parameters used in ‘Vesicle traffic’.

Symbol	Description	Equation
c^*^	Non-dimensional concentration, i.e., concentration *c* normalized by characteristic concentration of the system *C_o_*; c^*^≡c/C_o_	3
*D_iff_*	Diffusion coefficient of GTPase at the membrane	2
*h*	Total delivery rate of GTPase (GDI mediated, independent and exocytosis)	1
*h_w_*	Net delivery rate of GTPase within the delivery window (delivery minus removal)	1
*L*	Characteristic length of the sytem	1, 2
*L_w_*	Characteristic length of delivery window	1
*m*	Total membrane dissociation rate of GTPase (GDI mediated, independent and endocytosis) outside of the delivery window	2
x^*^	Non-dimensional variable for length, i.e. position *x* normalized by characteristic length of the system *L*; x^*^≡x/L	3
*ρ_del_*	Non-dimensional parameter; ratio between localized and global delivery of GTPase to membrane	1
*ρ_rem_*	Non-dimensional parameter; ratio between removal of GTPase from membrane and its diffusive flux	2

**Table 2:** Variables and parameters used in lumped and detailed models.

Symbol	Description	Equation
*Eff*	Concentration of effector proteins	4,5,15,16
*GDI*	Concentration of GDI molecules	4–7, 15,16
*k_i−_*	Unbinding rate of reaction number *i*	4–10
*k_i+_*	Binding rate of reaction number *i*	4–10
k^*^_i+_	Binding rate times concentration (of GDI when *i* = 1, 1L, 3, 3L; of effector when *i* = 5)	4–10
*K_Di_*	Dissociation parameter, ratio *k_i−_*/*k_i+_*	5,12
K^*^_Di_	Non-dimensional dissociation parameter, ratio *k_i−_*/k^*^_i+_	11
*K_DGDI_*	Non-dimensional dissociation parameter between cytosolic GTPase and GDI	8
*K_Dm_*	Non-dimensional dissociation parameter between cytosolic membrane and cytosolic GTPase (which is GDI free)	9
*k_offAp_*	Apparent dissociation rate between GTPase and membrane	4, 5, 16
*R_i+_*, *R_i−_*	Binding and unbinding rates of reaction number *i* for detailed model	6, 15,16
R^*^_i+_	Binding rate times concentration (of GDI when *i* = 3; of effector when *i* = 5)	15
*r0*	Fraction of GTPase at the membrane (number of molecules bound to the membrane divided by total number of molecules in the cell)	13
*r0_f_*	Fraction of GTPase at the membrane that is free from GDI (number of molecules bound to the membrane that are free from GDI divided by total number of molecules in the cell)	14
*Rho_L_*	Lumped concentration of RhoGTPase (includes GDP and GTP bound, and interaction with effector proteins)	13,14
*Sfc*	Membrane surface area	10
*Vol*	Cytosolic volume	10
*ρ_GDI_*	Non-dimensional parameter; ratio *K_D3L_*/*K_D1L_* or K^*^_D3L_/K^*^_D1L_; represents the impact of membrane localization to the affinity between GTPase and GDI.	11
*ρ_m_*	Non-dimensional parameter; ratio *K_D4L_*/*K_D2L_*; represents the impact of GDI on the affinity between the GTPase and the membrane	12
*ρ_Eq_*	Parameter used in detailed balance only, *ρ_Eq_* = *ρ_m_* = *ρ_GDI_*	13,14
( )*_m_*	Membrane bound species/complexes	4, 6, 7, 13–16
( )*_c_*	Cytosolic species/complexes	4, 6, 7, 13–16

**Table 3:** Variables and parameters used in ‘Application to Rac cycling in pancreatic β-cells’.

Symbol	Description	Equation
*A*	Coefficient representing increase in phosphorylation rate of GDI upon glucose stimulus	S24
*B*	Coefficient representing increase in binding rate between cytosolic Rac and plasma membrane due to active phospholipase D	S25
*C*	Coefficient representing increase in binding rate between cytosolic Rac and granular membrane due to active phospholipase D	S26
*kon_M_*	Binding rate between cytosolic Rac and plasma membrane	S25
*kon_Gr_*	Binding rate between cytosolic Rac and granular membrane	S26
*PLD_mi_*	Phospholipase D1, membrane bound and inactive	S23
PLD^*^	Phospholipase D1, membrane bound active	S23
pG	GDI phosphorylation rate	S24
sGDI	Concentration of GDI that is serine phosphorylated	S21,S22
( )_t_	Cytosolic GTPase or complex concentration at time t	S21,S22

In Section Vesicle traffic we first discuss this GDI independent cycling mechanism. We show that vesicle traffic occurs at much slower rates than GDI mediated membrane dissociation of GTPases. However, it has been hypothesized that localized traffic may contribute to polarized membrane distribution of GTPase, as in budding yeast. We present a dimensional analysis of the parameters involved in GTPase distribution in yeast and compare our analysis to recently published data [21], [22].

It has been shown that *in vitro* GTPases dissociate from membranes in absence of GDI at rates that may be as fast as in presence of GDI [23]. In the Section GDI and *k_offAp_* we analyze the contribution of GDI mediated and independent mechanisms to the apparent membrane dissociation rate of GTPases. We identify parametric regions where the apparent membrane dissociation rate is insensitive to GDI concentrations. This demonstrates the importance of considering the parametric region corresponding to a physiological system of interest.

The relative contributions of GDI dependent vs. independent cycling will also impact the fraction of GTPase at the membrane, a common experimental observable. In addition, a fraction of the membrane bound GTPase may be inert due to interaction with GDI. The analysis of the fraction of GTPase associated with the membrane is therefore the focus of Section Lumped model. We find that the kinetic rates of a simplified model can be lumped into the non-dimensional parameter *ρ_Eq_*, representing the degree of contribution of GDI to the membrane cycling of the GTPase. The results of this model are presented in charts, to visualize GTPase distribution as a function of GDI concentration and affinities between GTPase and GDI, and GTPase and membrane.

However, in order to use these charts, one must know the parameter *ρ_Eq_* and the regions of the chart that applies to the system of interest. In the Section Detailed model we build a model which includes nucleotide cycling. This model is the most appropriate for extraction of kinetic parameters. It successfully reproduces the cycling of Rac in NIH3T3 cells [15].

Finally, in the Section Example we illustrate another application of this modeling framework. We use the results from the Sections Lumped model and Detailed model to analyze the glucose stimulated Rac redistribution in pancreatic β-cells.

### Vesicle traffic

GTPases have been shown to localize to vesicles, and endocytosis and exocytosis can be considered as potential pathways to regulate the amount of GTPase at the plasma membrane. Proteins that are tightly bound to membranes are trafficked by fusion and scission of vesicles only. But small GTPases are able to diffuse in the cytosol, which therefore constitutes an alternative mechanism.

The first question we ask is: which is the fastest pathway for plasma membrane bound GTPases to reach the vesicular membrane? a) via endocytosis, or b) via dissociation from the plasma membrane, cytosolic diffusion (usually bound to GDI) and finally binding to the vesicle membrane. A simple argument demonstrates that cytosolic diffusion is the likely dominant pathway. For mammalian and yeast cells, recycling rates of membrane due to endocytosis lie on the timescale of 10^−4^/s [22], [31]. This number is obtained multiplying the rate of endocytosis (number of vesicles per time) times surface area of a single vesicle divided by the surface area of plasma membrane. Note that the GDI dependent removal of GTPase from the membrane is on the order of 10^−2^/s or higher [15], [21], [23]. This means that the kinetic term for traffic becomes important only if the concentration of GTPase in vesicles is at least one order of magnitude higher than in the plasma membrane. By conservation of membrane area, exocytosis is expected to have contributions of the same time scale as endocytosis when traffic is evenly distributed along the plasma membrane. This analysis in confirmed by a more detailed computational model [32]. This means that if a vesicle is able to sustain higher concentration of GTPase (molecules/µm^2^) than the plasma membrane, either the dissociation rate of GTPase from the vesicle to the cytosol is lower than from the plasma membrane to the cytosol, or the association rate from the cytosol to the vesicle is higher than to the plasma membrane. The lipid composition of the vesicular membrane, for example, may promote the higher affinity for GTPase. But this analysis assumes that the plasma membrane has uniform vesicle trafficking. So a second question may now be posed: can localized delivery, as may pertain in yeast [21], of vesicles with high concentration of GTPases generate a concentration polarization on the plasma membrane?

The answer depends on the balance of three rates: a) the net rate of delivery of vesicles to a localized region of the membrane (delivery window); b) the diffusion of GTPase to the other regions of the plasma membrane; and c) the removal rate out of the delivery window. The only mechanism fighting against polarization is the lateral diffusion of GTPase in the membrane. Using dimensional analysis, we now show that even if the rate for localized delivery of GTPases is infinitely high, the concentration gradient can only be sustained if the removal rate (out of the delivery window) is high enough to overcome the effect of lateral diffusion.

The parameters necessary for the analysis are the characteristic length of the delivery window *L_w_*, the characteristic length of the system *L*, the net delivery rate in the window *h_w_*, the delivery rate out of the window *h*, the first order rate constant for removal from the membrane outside of the window *m* and the lateral diffusion coefficient of the GTPase in the membrane *D_iff_*. These parameters are lumped into two non-dimensional numbers *ρ_del_* and *ρ_rem_*:
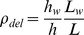
(1)

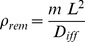
(2)
*ρ_del_* represents the ratio between localized and global delivery of proteins. *ρ_rem_* represents the ratio between the rate of removal of protein from the membrane and its membrane diffusive flux. The equation for conservation of mass for the non-dimensionalized concentration of GTPase at the membrane c^*^ is a function of these two ratios:
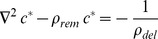
(3)The boundary condition for flux normal to the interface with the delivery window is −1 (see Text S1 and Eqs. S1–S7 for details). When *ρ_del_*<<1, the localized delivery is insufficient to generate a gradient regardless of *ρ_rem_*. However, *ρ_del_*→∞ is not sufficient to maintain a concentration gradient. It is also necessary that *ρ_rem_* is on the order of 1 or larger. The solution for the 1D problem is plotted in Fig. S1 in Text S1.

A series of numerical simulations of a model considering stochastic vesicle traffic in polarized yeast have been published [22], corresponding to the same problem in spherical coordinates. This study assumed negligible delivery outside of the window (*h = 0,ρ_del_*→∞), and the reference value for *ρ_rem_* was on the order of 10^−1^ (*m* = 1.7×10^−4^, and the characteristic length of the cell is its diameter, *L* = 5 µm). The simulations were performed considering *m* to be due to vesicle endocytosis alone. Increasing the net delivery rate *h_w_* (via concentration on exocytic vesicles or frequency of exocytosis) increased the membrane concentration of Cdc42, but did not sustain polarization at steady state. This is consistent with the fact that *ρ_del_* remained unchanged (*ρ_del_*→∞). However, polarization became noticeable as *ρ_rem_* is increased by one order of magnitude (via decreased *D_iff_* or increased endocytosis from membrane regions outside the window).

In contrast, *ρ_rem_* values based on iFRAP measurements in yeast [21] range from 13 to 150 (removal rates *m* ranges from 0.02 to 0.22 s^−1^). The lowest value corresponds to experimental conditions that completely eliminate the contribution of rdi1 mediated cycling to *m*. Polarization does occur, consistent with the size of the parameter *ρ_rem_*. It is important to note that mutations abrogating the binding between Cdc42 and rdi1 still resulted in *m* values two orders of magnitude higher than that estimated to be due to endocytosis [22], suggesting that direct dissociation of Cdc42 from the membrane dominates under these conditions. Thus, our analysis shows that the measured values of *m* in polarized yeast are sufficient to generate a gradient in Cdc42 concentration. The simulations in [22] confirm that if the removal rate *m* is due to vesicle traffic alone, no polarity is established. However, polarity would be facilitated by considering the removal rate due to a third mechanism that is rdi1 and vesicle independent. This possibility has not been explored in yeast so far.

This short analysis is built on top of the series of detailed models that have contributed to understanding yeast polarity. The new insight is that we reveal the two non-dimensional numbers that dictate the behavior of the system, *ρ_del_* and *ρ_rem_*. There is a single solution for equation (3) for a given pair *ρ_del_* and *ρ_rem_*. However, there is an infinite combination of parameters that would result in the same pair *ρ_del_* and *ρ_rem_*.

### GDI and *k_offAp_*: Contribution of GDI to the apparent membrane dissociation rate

**Figure 1:**
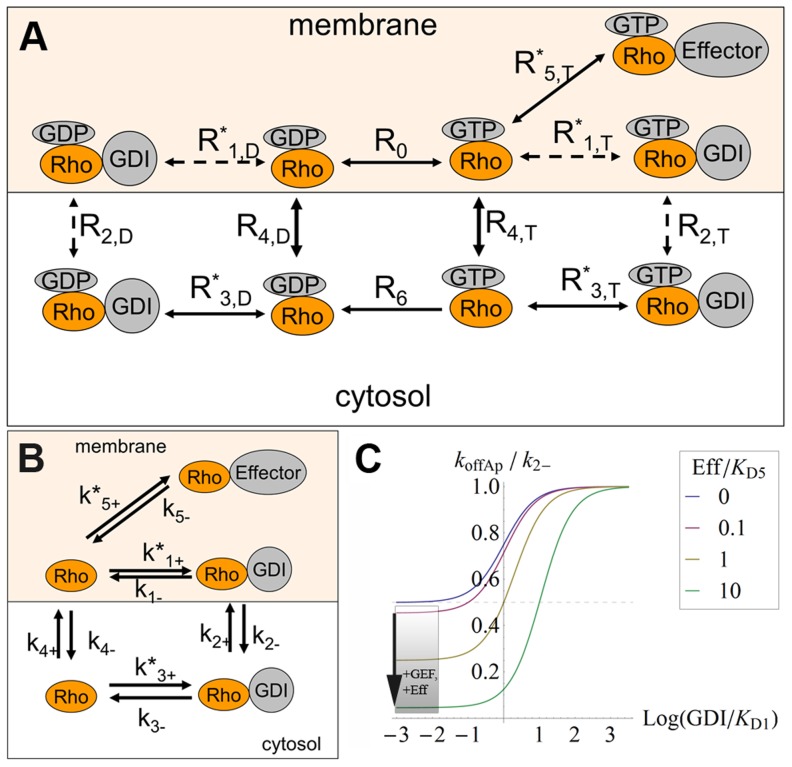
Models for GTPase membrane cycling. The asterisk represents the product between binding rates and concentration of GDI (subscripts that carry the numbers 1 and 3) or effector proteins *Eff* (subscript 5). In all models presented, the numbers used as subscripts for the cycling rates are consistent: 1 stands for interactions between membrane bound GTPase and GDI; 2 for membrane cycling of the complex GTPase-GDI; 3 for interactions between cytosolic GTPase and GDI; and 4 for membrane cycling of GTPase free from GDI. A. Detailed model. B. GDI dependent and independent GTPase cycling. Rates with subscript “+” represent binding (to GDI, effector proteins or membrane). C. Apparent membrane dissociation rate (*k_offAp_*) normalized by the GDI mediated dissociation rate *k_2−_* as a function of *K_D1_*, *K_D5_*, *GDI* and *Eff*.

Membrane dissociation of GTPases via an unexpected rdi1 (or GDI) independent mechanism that occurs much faster than vesicle traffic would not be unique for Cdc42 in yeast. The membrane dissociation rate of Rac in NIH3T3 cells is independent of GDI concentration and is up to two orders of magnitude faster than endocytosis rates [15], [33]. In addition, GDI independent membrane dissociation of GTPases has been shown *in vitro* [11], [23]. The complete model for membrane cycling and activation of GTPases is displayed in Fig. 1A (explained in Section Detailed model). Note that the interaction with GDI inhibits the nucleotide exchange [11], [34]. We assume that when the GTPase is bound to effector proteins the complex does not dissociate from the membrane (or dissociates at much slower rate than the GTPase alone). This assumption is justified by the fact that membrane-localized activation of GTPases results in local cytoskeleton reorganization [35]. The corresponding *in vivo* rates have not been determined because the proper identification and measurement of all different states of the GTPase is an experimentally daunting challenge. However, the analysis presented here shows how to combine the use of currently available experimental techniques with a simplified “lumped” model, depicted in Fig. 1B. The main objective is to identify the relevant pathways for removal of GTPase from the membrane.

The common observation for all experiments is that increased GDI concentration results in decreased fraction of GTPase at the membrane. The question we ask is whether GDI accelerates the removal rate of GTPase from the membrane (as shown for Cdc42 in yeast [13]) or simply acts as a buffer, preventing the binding of GTPase to the membrane (as proposed in [23]). The measurable quantity that will help answer this question for *in vivo* experiments is the apparent membrane dissociation rate of GTPase *k_offAp_*.

We can assess the relative contributions of the GDI-independent and dependent membrane dissociation mechanisms to *k_offAp_* by analyzing experiments in which GDI concentrations and effector protein concentrations are varied. If the experiments reveal increased *k_offAp_* with increased GDI concentration, then the complex GTPase-GDI represents a significant pathway for membrane removal of GTPase. Even if the membrane dissociation rates for free and GDI-bound GTPase are identical (as shown in the *in vitro* experiments for Cdc42 [23]), by increasing the concentration of effector proteins, a dependence of *k_offAp_* on GDI concentration should become explicit (Fig. S3 in Text S1). In contrast, the independence of the *k_offAp_* on GDI concentration would reveal that the primary mechanism of removal of GTPase from the membrane is GDI independent.

In Fig. 1B the GTPase binds or unbinds the membrane while free or bound to GDI. The apparent dissociation rate *k_offAp_* is the experimental observable (for example, from FLIP experiments; [15], [36]). The dependence of *k_offAp_* on membrane dissociation rates *k_2−_* and *k_4−_* (for membrane bound GTPase in complex with GDI or free, respectively) and GDI concentration:
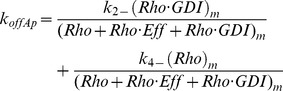
(4)Where the small RhoGTPase is represented by *Rho*, *Eff* represents the effector proteins, binding between two proteins is represented by a dot, and the subscript *m* represents membrane bound species.

Under equilibrium conditions, Eq.(4) can be rewritten:
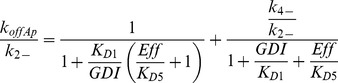
(5)The dissociation parameters *K_Di_* are defined as the ratio *k_i_*_−_/*k_i+_*, with subscript *i* corresponding to each numbered reaction in Fig. 1B. We call it ‘parameter’ rather than ‘constant’ since the rates can be modulated during signaling. Because the fastest membrane dissociation rate is believed to be GDI mediated, it is convenient to look at the non-dimensional ratio *k_offAp_*/*k_2−_* This is equivalent to normalizing *k_offAp_* relative to its maximal value in the presence of saturating GDI. Figure 1C shows a plot of *k_offAp_/k_2−_* as a function of the ratio between concentration of free GDI and *K_D1_* for several values of concentration of effector protein divided by *K_D5_*. Changes in GDI expression levels are showed in the x-axis. Increased concentrations of effector proteins are depicted by different curves (or alternatively, increased affinity between GTPase and effector protein, due GTPase activation). For this example, the GDI independent dissociation rate was taken as one half the GDI mediated dissociation (*k_4−_* = 0.5 *k_2−_*); as shown in the supplementary material, this assumption does not affect the qualitative arguments developed here (Fig. S3 in Text S1). Figure 1C shows that the maximum *k_offAp_* equals *k_2−_* when the membrane bound population of GTPase is all GDI bound. In the absence of effector proteins (or for a GTPase unable to bind the latter), *k_offAP_* cannot be reduced below *k_4−_* (dashed line). This limiting value is reached either when the concentration of GDI is negligible, or the probability of the membrane bound GTPase to bind GDI is negligible. Importantly Fig. 1C reveals that *k_offAp_* can remain unchanged over a wide range of GDI expression levels when the membrane bound GTPase is either all bound to GDI (*K_D1_<<GDI*), or, at the other extreme, has negligible affinity for GDI (shaded region)Interestingly, and somewhat counterintuitively, in the regime where *k_offAp_* is sensitive to GDI (*K_D1_*≅*GDI*), increasing the concentration of effector proteins will accentuate this sensitivity. One extreme case is when the membrane dissociation rates for GDI free or bound GTPase are identical (as shown for Cdc42 *in vitro* [23]). As shown in Fig. S3a in Text S1, in absence of effector proteins, increasing or decreasing the GDI concentration will not perturb *k_offAp_*. However, for higher levels of effector proteins, *k_offAp_* will be sensitive to GDI concentrations, in the concentration range *GDI*≅*K_D1_*.

Data for *k_offAp_* vs. *GDI* will help identify which region of Fig. 1C pertains to the experimental conditions and the regime within which the particular GTPase operates. Such experiments can be developed by expressing different amounts of GDI, effector proteins, or mutants that will result in different affinities between GTPase and GDI or effector proteins. An immediate application of this analysis is the specific example of Rac in NIH3T3 cells, using the experimental data published by Moissoglu and colleagues [15]. These experiments are further described in the Section Detailed model. Briefly, wild type (wt) Rac or the constitutively active G12VRac were co-expressed with different levels of GDI in cultured NIH3T3 cells, and *k_offAp_* was measured. The experimental results show that *k_offAp_* is independent of GDI concentrations both for constitutively active or wild type Rac However, the *k_offAp_* in cells expressing G12VRac was tenfold lower than in cells expressing wt Rac. Expression of G12VRac would produce similar behavior as activation or overexpression of a GEF, since both conditions result in increased net affinity between the GTPase and effector proteins. The region of Fig. 1C that reproduces both the independence on GDI concentration and dependence on GTPase activity level is highlighted by the shaded box. We do not know the *k_2−_* for this system, so it is not clear whether Fig. 1C or one of the other plots in Fig. S3 in Text S1 would be the best representation of the experiment. However, all show the same progression of *k_offAp_* as the effector activity increases in the region *K_D1_*>>*GDI*. This example will be revisited in Section Detailed model. The analysis suggests that the physiological range that describes Rac cycling in NIH3T3 cells corresponds to *K_D1_*>>*GDI* and the function of GDI in this system is to act as a buffer, rather than accelerate the extraction of Rac from the membrane.

### Lumped model: membrane interactions between GTPases and GDI

**Figure 2:**
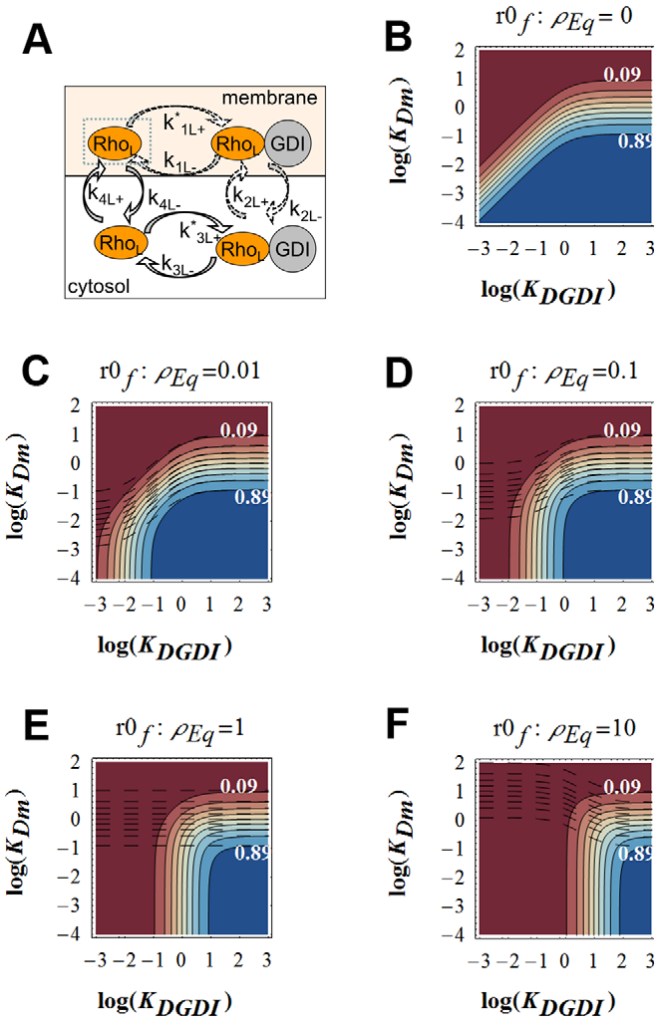
Model for analysis of fraction of GTPase at the membrane. A. Model for lumped variables and rates. The term in the dotted box includes effector bound GTPases. The dashed arrows represent the GDI mediated membrane cycling of GTPases. B–F. Fraction of GTPase at the membrane free from GDI as *ρ_Eq_* ranges from 0 to 10. The upper contour corresponds to 9% fraction at the membrane, while the lowest line represents 89%. Each pair of neighboring lines is 10% apart in membrane fraction. When *ρ_Eq_* = 0, all membrane bound GTPase is free from GDI (*r0* = *r0_f_*). The total fraction of GTPase at the membrane *r0* is represented by the dashed lines when *ρ_Eq_*>0.

The relative contribution of GDI free and bound GTPase to membrane cycling will also have consequences on the fraction of GTPase at the membrane. The main purpose of this section is to provide charts that relate membrane fraction of GTPases, the two cycling mechanisms and GDI concentrations. The results presented here will be used in the Section Example. We next build a model, Fig. 2A., which is appropriate for biological systems where both GDI mediated and independent mechanisms contribute to GTPase cycling. It can be used to assess how GDI activity, affinities between GTPases and GDI, or GTPases and membrane, each affect the fraction of GTPase that is membrane bound. Using the model, one can also predict how changes in the rates in Fig. 2A., either due to experimental manipulation or cell regulatory mechanisms, affect the translocation of the GTPase. The main issue is that it is not always possible to measure all the rates in Fig. 2A. However, we show that the eight rates in Fig. 2A can be replaced by only 3 parameters at thermodynamic equilibrium. These three parameters will uniquely determine two variables of interest: the fraction of GTPase at the membrane, and the fraction that is also free from GDI. The results are reported in the contour plots Fig. 2 (and Fig. S4 in Text S1). Because the solution is uniquely determined, any three measurements out of the five observables (three parameters plus two variables), will allow us to extract the remaining two unknowns.

In this model the nucleotide state and effector bound GTPases are collected into lumped variables, identified by the subscript L. In Fig. 2A, the variable Rho_L_ consists of the GDP and GTP and effector- bound forms. The relation between variables in Fig. 2A and Fig. 1A is presented:
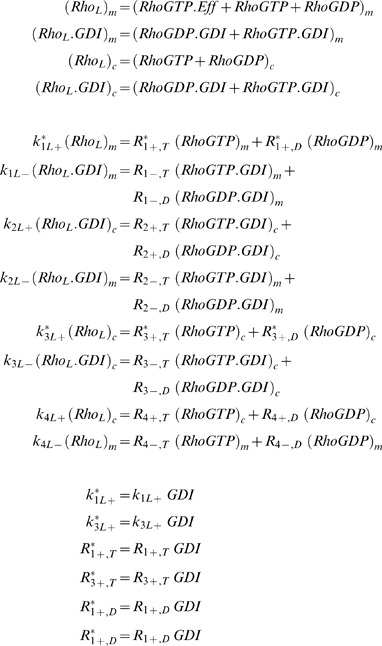
(6)The subscripts *c* and *m* represent cytosolic and membrane bound species, respectively. The steady state equations corresponding to Fig. 2A:
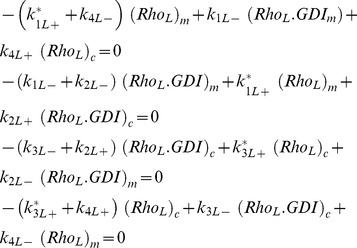
(7)It is convenient to define:
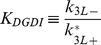
(8)

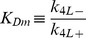
(9)
*K_DGDI_* represents the non-dimensional dissociation constant for cytosolic GTPase binding to GDI. *K_Dm_* represents the non-dimensional dissociation parameter between GTPase and the membrane, independent of GDI.

In most compartmental kinetic models involving fluxes to or from the membrane, the concentration of molecules is expressed relative to the volume of the cytosol. It is important to appreciate therefore that for a given surface density of a membrane species, the surface to volume ratio will scale all membrane fluxes. The ratio between membrane surface area *Sfc* to cytosolic volume *Vol* in the cell type of interest can therefore affect the association rate *k_4L+_*:
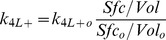
(10)Where the subscript ‘o’ represents the experimental conditions (e.g. an *in vitro* lipid vesicle assay) for which the membrane association constant *k_4L+o_* was obtained.

The relative contribution of GDI mediated GTPase cycling is best visualized using the coefficients:
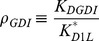
(11)

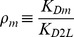
(12)The ratio *ρ_GDI_* smaller than unity means that the membrane bound GTPase is less likely to be GDI bound than the cytosolic GTPase. *ρ_m_* smaller than unit means that the GDI-bound GTPase has lower affinity for the membrane than the GDI-free GTPase.

The quantities of interest are the fraction of total GTPase bound to the membrane *r0*, and the fraction of GTPase at the membrane and free from GDI, *r0_f_*. They consist of ratios between the number of molecules at the membrane and total amount of GTPase in the cell (membrane plus cytosol). At thermodynamic equilibrium (a more restrictive condition than just steady state), the principle of detailed balance dictates that each reaction in Fig. 2A must have identical flux as its reverse reaction. It can be easily shown that for the equilibrium condition, *ρ_GDI_ = ρ_m_*. Taking *ρ_Eq_ = ρ_m_ = ρ_GDI_*, the solutions for *r0* and *r0_f_* are dependent on the dissociation parameters only:
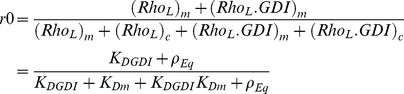
(13)

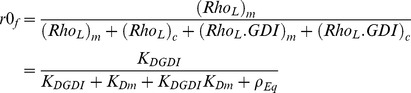
(14)
Equations (13) and (14) display the relationship between the five observables mentioned above: *K_DGDI_*, *K_Dm_*, ρ*_Eq_*, *r0* and *r0_f_*. For a system in equilibrium, measurement of any three of these will determine the remaining two. Figure 2 (and Fig. S4 in Text S1) shows the contour plots representing Eq.(14) for ρ*_Eq_* ranging from 0 to 10. Essentially, ρ*_Eq_* measures the extent to which GDI directly affects membrane-associated events in the overall mechanism of GTPase cycling. The upper contour corresponds to 9% fraction at the membrane, while the lowest line represents 89%. Each pair of neighboring lines is 10% apart in membrane fraction. When ρ*_Eq_* = 0, all membrane bound GTPase is free from GDI (*r0* = *r0_f_*). The total fraction of GTPase at the membrane *r0* is represented by the dashed lines when ρ*_Eq_*>0. Note that *K_DGDI_* is inversely proportional to GDI concentration (equations 6 and 8). Decreased *K_DGDI_* (high GDI concentrations) results in larger deviations between *r0* and *r0_f_*, increasing the amount of inert GTPase (GDI bound) at the membrane (difference between solid and dashed lines). These series of plots shows that in biological systems with large values of ρ*_Eq_* there is a large pool of inactive membrane bound GTPase due to interaction with GDI.

Clearly, a cell can utilize many mechanisms to modulate the reactions in Fig. 2A (i.e. phosphorylation at several sites of GTPase or GDI, nucleotide state, lipid composition and/or post-translational modifications). The contours in Fig. 2 (and Fig. S4 in Text S1) indicate the direction in parameter space that will produce the largest change in the membrane fractions *r0* and *r0_f_*.

As noted above, according to the principle of detailed balance, which pertains to systems in thermodynamic equilibrium, there can be no net flux through a cyclic reaction path; this dictates that *ρ_GDI_* = *ρ_m_*. For systems that do not reach thermodynamic equilibrium, the disparity between the coefficients *ρ_GDI_* and *ρ_m_* offer further insights into the role of GDI. Detailed balance does not need to hold in live cells, due to dynamic modulation of affinities (via phosphorylation states, membrane composition, etc…) or other factors that may perturb the system (production, degradation or other interactions). Nevertheless, significant deviations from the equilibrium implies that substantial energy needs to be fed into the system [37]. Steady state may still be achieved, however with a net flux through the cycle in Fig. 2A. The direction of the flux is determined by the second law of thermodynamics [38]. When *ρ_GDI_*>*ρ_m_*, the net flux flows clockwise, and GDI is promoting the removal of GTPase from the membrane. When *ρ_GDI_*<*ρ_m_*, it flows counterclockwise and GDI is promoting the delivery of GTPase to the membrane. Solving Eqs.(S14) in Text S1 for steady state will allow for generation of contour plots as in Fig. 2 (and Fig. S4 in Text S1). However, knowledge of the association and dissociation rates becomes necessary.

In summary, quantification of the relationship between *ρ_m_* and *ρ_GDI_* will determine the role of GDI in the system of interest. It is expected that during the short term response to a signal, the cell generates an inequality between *ρ_m_* and *ρ_GDI_*, using the GDI to promote delivery or removal of GTPase from the membrane. However, due to energetic cost, any long lived response to a given stimulus is most likely to be well described by detailed balance, where *ρ_m_* = *ρ_GDI_*.

### Detailed model: nucleotide and GDI dependent membrane cycling

**Figure 3:**
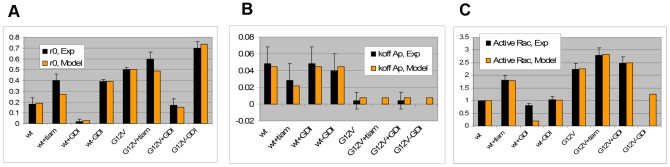
Comparison between experimental data “Exp”, black bars [**15**] and “Model”. A. Percentage of Rac at the membrane r0. B. Apparent membrane dissociation rate koff Ap (s^−1^). C. Total active GFP-Rac in the cell. The nomenclature for each of the eight experimental conditions is defined in Section Detailed model. Experimental measurements of koff Ap were not performed for 2 conditions and of Active Rac for one condition; the model predictions for those conditions are provided.

**Figure 4:**
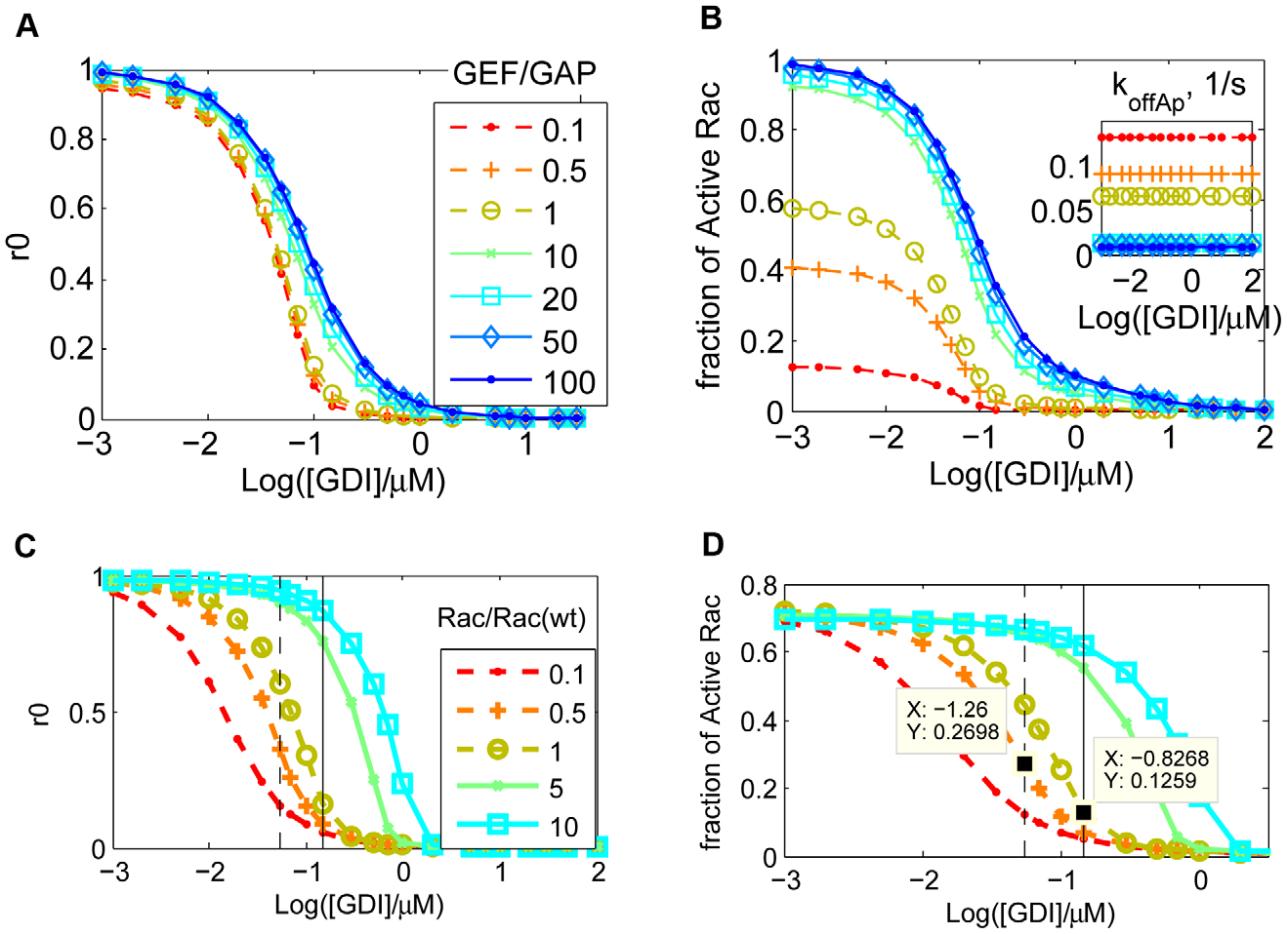
Dependence of fraction of GTPase at the membrane r0, membrane dissociation rate *k_offAp_*, and fraction of active Rac on GDI concentration. A–B. effect of GEF/GAP ratio. C–D. Effect of Rac concentration, relative to experimental condition ‘wt’. Vertical lines: GDI concentration for ‘wt’ (solid), and for ‘wt−GDI’ (dashed). While the fraction of active Rac doubled for ‘wt−GDI’, the total amount of active Rac is the same when Rac concentration is decreased by half (curve with crosses relative to open circles).

In order to use the results presented in the previous section, we must identify which chart is appropriate for the system of interest. Therefore, in this section, we use a detailed model (Fig. 1A) that includes nucleotide state and use it to extract kinetic parameters for RhoGTPase cycling. We apply this model to the experiments by Moissoglu and colleagues [15]. Using the analysis developed in the Section GDI and *k_offAp_*, we test the simplifying assumption that the main pathway for the membrane dissociation of Rac is GDI independent. Accordingly, the parameter values are optimized for this simplified model. But it is important to emphasize that the success of any parametric search depends on how well the topology of the model reproduces the system of interest [39]. Meaning that no matter how extensive the parametric search, an oversimplified system of equations will fail to reproduce the experiments. The simplification prior to optimization is necessary for two main reasons. First, the number of data points must be greater than the number of unknowns. Second, if the model includes reactions with negligible impact on observables an infinite number of parametric solutions will result in the same observable, making the parametric set of the model ‘non-identifiable’ [39]. Thus, the analytical study of Section GDI and *k_offAp_* serves as a guide for building a model that is consistent with experimental observations. The model is verified by the success of the parametric optimization (Fig. 3). The next step is validation. It consists of comparing the output of the model to experimental data that have not been used in the parametric search. We compare the results of the model (Fig. 4) with key features of GTPase systems: activation of GTPases (increased GEF activity) promotes translocation of GTPase to the membrane [18]; increased GDI results in removal of GTPase from the membrane [14]; depletion of GDI results in increased activity levels of GTPase [7], [40].

Moissoglu and colleagues developed a photobleaching method applied to live NIH3T3 cells in combination with a mathematical model in order to extract the dissociation rate of GFP-Rac from the cell membrane [15]. Briefly, photobleaching of the whole cell exclusive of a narrow area at the edge was performed; the fluorescence decay in the unbleached area provided a measure of GFP-Rac dissociation and diffusion. The diffusion coefficient for GTPase in the membrane was extracted by repeating the experiments with different widths of unbleached region. The change in fluorescent GFP-Rac was used to compute the GTPase membrane dissociation rate constants (*k_offAp_*), corrected for membrane diffusion. The detailed error analysis of the method is further explored in [36]. Experiments were performed for cells expressing two forms of GFP-Rac: of the wild type (wt) and a constitutively active (G12V) mutant. The experiments were repeated for cells co-expressed with different amounts of GDI. The experimental data reveals plots of *k_offAp_* versus GDI concentration (similar to the one presented in Section GDI and *k_offAp_*), showing that the *k_offAp_* in this system is completely independent of GDI expression levels. However, co-expression of wt and a GEF decreases *k_offAp_*; in cells expressing G12V the *k_offAp_* is further decreased by one order of magnitude compared to cells expressing wt.

These results are consistent with our analysis in Section GDI and *k_offAp_* (see shaded area of Fig. 1C). An additional complication is that the membrane dissociation rates for GDP or GTP bound Rac does not need to be identical. In fact, the cell could benefit from having the active GTPase remain at the membrane for longer time than the inactive. As a consequence, *k_offAp_* may be further decreased due to the combined effect of increased affinity for effector proteins and decreased net dissociation rate. We allow the dissociation rates for GTPase bound to GDP or GTP to be different in the parametric search. The fact that GDI was not found in the membrane fraction of NIH3T3 cells reinforces the conclusion that the system is cycling under the conditions (*GDI*/*K_D1_*)∼0 in Fig. 1C.

We now describe the model. RhoGTPases that are at the membrane and not associated with GDI, cycle between the active GTP bound and inactive GDP bound states due to GEFs and GAPs. Cytosolic GAP activity has also been reported [41]. The GTPase bound to either nucleotide is subject to the reactions in Fig. 1A: it may bind to GDI while at the membrane or in the cytosol, and the GTPase or complex GTPase-GDI binds and unbinds the membrane. When the GTPase is membrane bound, active and free from GDI, it may bind to effectors. Based on the discussion above and in Section GDI and *k_offAp_*, we neglect the reactions associated with the Rho-GDI in the membrane (the dashed reactions in Fig. 1A.), and check if this topology is a good representation of the system. The simplified system of equations:
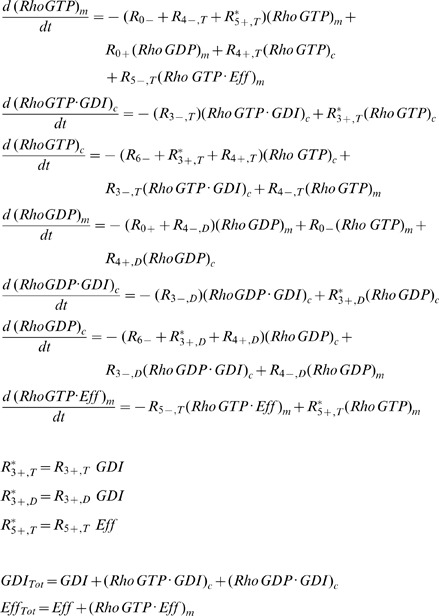
(15)The experimental value for apparent dissociation rate using the photobleaching method can be expressed in terms of the remaining rates:
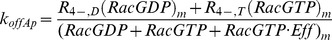
(16)Where *R_4−,D_* and *R_4−,T_* are the membrane dissociation rate constants for inactive and active Rac, respectively.

The other measurements provided by Moissoglu et al. [15] were the percentage of Rac in the membrane using cell lysates, *r0*, and the total amount of active GFP-Rac (including membrane and cytosol). Eight experimental conditions were considered and are labeled here as follows: ‘wt’ corresponds to transfection of GFP-wtRac; ‘wt+tiam’, GFP-wtRac cotransfected with the GEF Tiam1; ‘wt+GDI’, GFP-wtRac cotransfected with GDI; ‘wt−GDI’, GFP-wtRac in GDI knockdown cells, and the same four experimental conditions repeated for GFP-G12VRac (constitutively active) transfection instead of GFP-wtRac are labeled ‘G12V’, ‘G12V+tiam’, ‘G12V+GDI’ and ‘G12V−GDI’ respectively. Measurements of total active GFP-Rac were normalized by the result for the control experiment ‘wt’. The cells were reported to have a surface to volume ratio of 0.524/µm.

Model simplifications and the methodology used for the parametric search are presented in the supporting material Text S1.

The model (solid arrows in Fig. 1A) is able to reproduce the quantitative behaviors of cycling and activation of Rac in cultured NIH3T3 cells (Fig. 3), thus validating the model. We find the membrane dissociation rate for Rac-GDP to be one order of magnitude higher than for active Rac. Figure 3 corresponds to cells with endogenous GDI concentrations of 0.14 µM, GFP-Rac of 0.05 µM, total effector concentration 0.5 µM, basal membrane GEF/GAP is 1.9, with 3.25 fold increase by Tiam. The ratio between the dissociation constants between cytosolic GDI and cytosolic Rac bound to GDP versus bound to GTP is 0.99. This value differs from what has been previously reported in experiments performed using solution of low ionic strength [8]; however, the importance of physiological ionic strength in measurements of GDI-RhoGTPase binding has been demonstrated in similar systems [9]. The dissociation parameter between cytosolic Rac and GDI is 1.3×10^−4^ µM. The association rate between Rac and the membrane is 2.84/s and dissociation rates between GDP and GTP bound Rac and the membrane are 0.15/s and 0.011/s, respectively. Note however, that free (unbound to GDI) Rac seems to be tightly bound to the membrane (with membrane dissociation parameter between 0.004 and 0.05). The effective dissociation constants between cytosolic GDI and active or inactive membrane bound Rac are also one order of magnitude apart ((*R_3−,T_ R_4+,T_*)/(*R_3+,T_ R_4−,T_*) = 34 nM and (*R_3−,D_ R_4+,D_*)/(*R_3+,D_ R_4−,D_*) = 2.5 nM, respectively), and within the range measured for Cdc42 (1–30 nM), [42], [43]. The parameters derived from the remarkable fits between experiment and model in Fig. 3, serve as a basis for our further exploration of the Rho GTPase system. Further results can be found in the supporting material Text S1.

While these parameters are within the expected physiological range, it is important to caution that they are somewhat sensitive to the basal GEF/GAP activities as well as the intracellular concentrations of endogenous GDI and the levels of effectors, which, as discussed above, are approximated to be in the same concentration range as the RhoGTPases. Another approximation is that the concentration of endogenous Rac was considered constant upon transfection of GFP-Rac, Tiam1 and GDI, and reduced by half upon GDI knockdown. In the same manner that GDI knockdown may promote Rac degradation [7], [15], [40] the endogenous Rac levels might have also been perturbed by the different GFP constructs.

To illustrate the sensitivity of the output functions to these approximations, we compute the effect of variations in the GDI concentration and the ratio between GEF and GAP using the optimized parametric set (Fig. 4). Consistent with the experimental studies, the *k_offAp_* (Eq. 16) has negligible dependence on GDI concentration while it is very sensitive to the GEF/GAP ratio (inset in Fig. 4B). In very low concentrations of GDI, approximately 5% of Rac will leave the membrane and become cytosolic. The effector concentration is able to increase the amount of active Rac in the absence of GDI (for GEF/GAP = 1, the fraction of active Rac is higher than 50%). However, at high GDI concentrations most of the Rac is cytosolic and inactive, even for very high GEF activity. Thus, Figure 4 A–B makes explicit that knock down of GDI results in translocation of GTPase to the membrane, and increased net activity; the subsequent degradation of Rac would act as a compensatory mechanism.

Importantly, Figure 4C–D reconciles the experiments for GDI knock-down ‘wt−GDI’ in fibroblasts [15] with more recent experimental data [7]. In the former, the same amount of active Rac is reported for ‘wt−GDI’ and ‘wt’ experiments. In contrast, the latter reference reports that depletion of GDI results in decreased GTPase expression and increased GTPase activity. Figure 4C–D reports *r0* and fraction of active Rac as a function of GDI concentration for different levels of GTPase. The reference curve (open circles) corresponds to the concentration of Rac as in the experimental conditions ‘wt’. The solid vertical line marks the GDI concentration for the same experiment. Decreasing the expression level of Rac alone would decrease the fraction of Rac at the membrane and its activity level (curve with plus sign). The dashed vertical line marks the GDI concentration for the experiment ‘wt−GDI’. The solid squares show the points corresponding to GDI and Rac concentrations for ‘wt’ and ‘wt−GDI’ in Fig. 4D. The fraction of active Rac is doubled (from 13% to 26%), while the concentration of Rac is decreased by half. In addition, the model predicts that Rac degradation prevented a further increase of up to 44%.

We learn from this model that membrane cycling of Rac in NIH3T3 cells at steady state can be represented by a model where Rac dissociates from the membrane prior to its binding to GDI. The model topology is verified by the results in Fig. 3. For a fixed GDI concentration, activation of a GEF results in the translocation of Rac from the cytosol to the membrane. The effect of the GEF is to reduce the dissociation of Rac from the membrane (reduce *K_Dm_*), resulting in a larger fraction of membrane bound Rac and a smaller fraction of cytosolic GDI bound Rac, consistent with *in vitro* experiments [18]. The knockdown of GDI results in translocation of GTPase to the membrane. The translocation alone promotes the activation of Rac, due to colocalization between the GEF and GTPase at the membrane. Furthermore, the degradation of Rac functions as a negative feedback, attenuating its hyperactivity.

### Example: Application to Rac cycling in pancreatic β-cells

In this section we illustrate how the parametric plots presented in Section Lumped model can be used to infer which modulatory events downstream of a signaling cascade are responsible for the experimental observations. We choose Rac cycling between cytosol and membranes (plasma and granular) in pancreatic β-cells during the second stage of insulin secretion upon glucose stimulus. This choice is based on its significance in diabetes and the ample availability of relevant experimental data regarding Rac [12], [17], [44]–[51]. Despite these many studies, thus far, it has not been possible to experimentally determine whether Rac translocation to the membrane is due to its decreased affinity for serine phosphorylated GDI (sGDI) alone, or whether a second signal promoting increased affinity between Rac and the plasma membrane is also necessary [52]. We show that for a cell with the plasma and vesicular membranes, as in β-cells, the observed translocation of Rac can only be reproduced if the stimulus promotes both decreased affinity for GDI and increased affinity for the plasma membrane.

Because Rac is localized both in plasma and vesicular membranes in β-cells [50], the ratio of membrane surface to cytosolic volume is much larger than the ratio that pertained to our analysis of fibroblasts in Section Detailed model. The total surface area of vesicles is seven fold the plasma membrane area, while the maximum exocytosis rate for a membrane bound molecule is 2.4×10^−4^/s [45], [48], [49]. Note that the cycling rate of GTPase between plasma and vesicular membranes via the cytosol is two orders of magnitude faster than via vesicle fusion and scission [15]. This leads to a model simplification: the vesicular traffic of GTPase does not need to be modeled, simply the total surface area of vesicles. Ideally, the same experimental procedures obtained for NIH3T3 should be repeated for β-cells. Unfortunately this data is not currently available. We use the kinetic data obtained in the Section Detailed model, the geometric data on the β-cells (surface area of plasma and vesicle membranes and intracellular volume), and the membrane fraction of Rac prior and 20 minutes after glucose stimulus to locate relevant regions of our contour plots.

Although the overall system is still evolving at 20 min, the time scale of the glucose signaling cascade (several minutes) justifies a quasi-steady state approximation (the timescale for Rac cycling is seconds). The translocation of Rac in β-cells is attributed to two mechanisms: phosphorylation of GDI by Pak1, increasing *K_DGDI_* [12], and activation of Phospholipase D1, decreasing *K_Dm_* [51]. While the former mechanism was proved essential, the contribution of the latter has not been quantitated. However, it is known that inhibition of phosphatidic acid production obliterates the first stage of insulin secretion, which is upstream from the *K_DGDI_* modification [52]. We next use the analysis of the contour plots to highlight the relevant properties of the system. More detailed description of Rac cycling in β-cells can be found in the supporting material Text S1.

**Figure 5:**
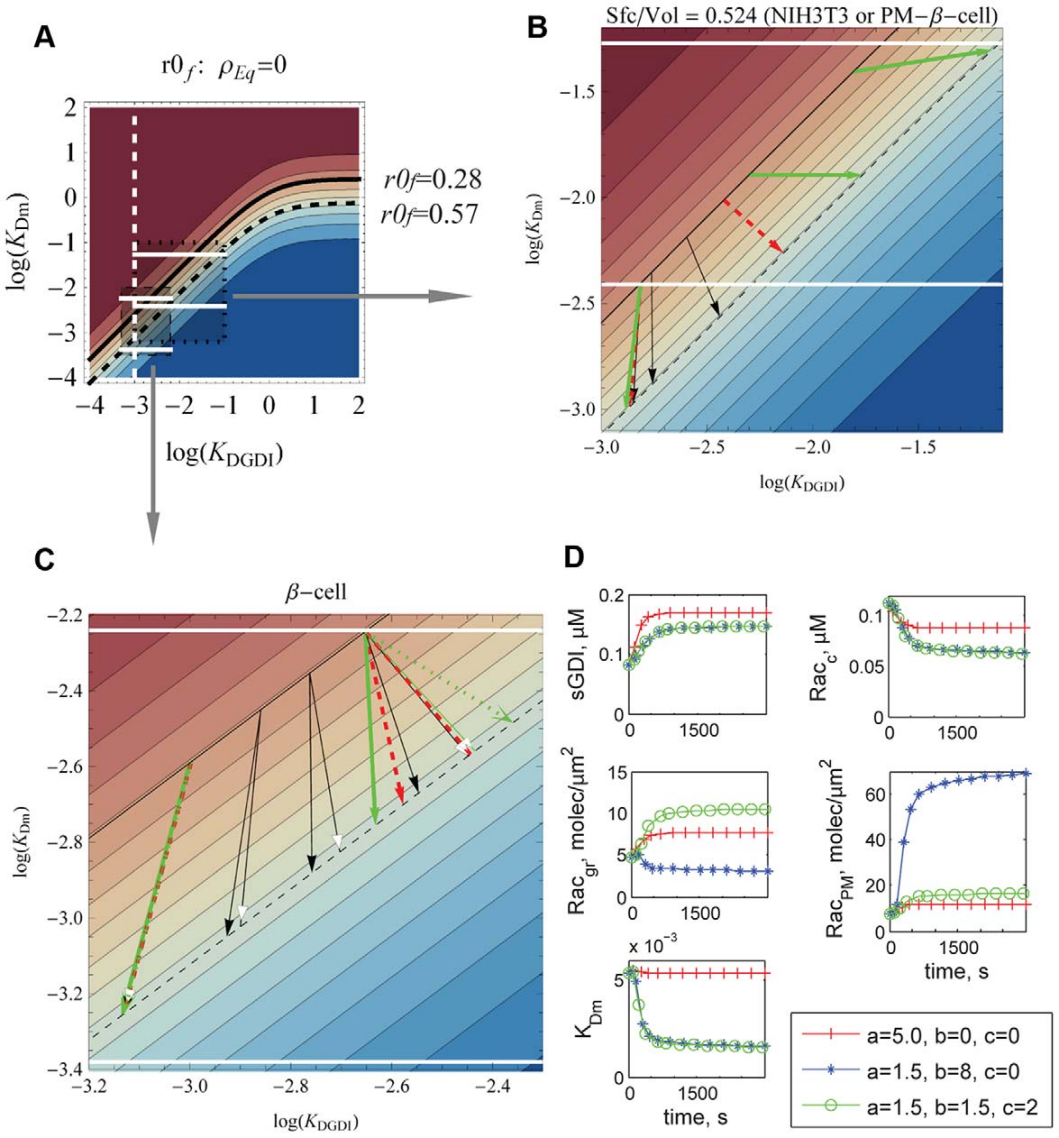
Membrane fraction of Rac before and after increase in sGDI due to stimulus. A–C. Horizontal lines delimit *K_Dm_* for minimum and maximum GEF/GAP based on values in Section Detailed model, for a cell with (B) Sfc/Vol = 0.544/µm, corresponding to NIH3T3 cells or the surface area of only the plasma membrane of β-cell, or (C) Sfc/Vol = 4.8/µm, corresponding to the surface area of both plasma and granular membranes of the β-cell. At time 0, *r0_f_* = 0.28 (solid bold curve), and at 20 minutes *r0_f_* = 0.57 (dashed black curve). Arrows represent effective trajectories that satisfy the 70% increase in sGDI and 40% decrease in cytosolic Rac. Arrow type for fold increase in dissociation constant between Rac and sGDI in comparison to unphosphorylated GDI (see text): 5, solid; 10, dashed; 100 (B) and 1000 (C), bold (solid and dotted). For solid arrowheads, GDI bound to Cdc42 was considered inert. White arrowheads consider the effect of phosphorylation of GDI bound to Cdc42. All arrows, 0.15 µM cytosolic Cdc42 bound to GDI, but for dotted, 0.25 µM. D) Transient translocation of Rac between cytosol (Rac_c_), plasma (Rac_PM_) and granular membranes (Rac_gr_) due to change in K_DGDI_ only (cross), plus increased affinity for plasma membrane (star, b>0) and granular membrane (circle, c>0).

First, we must identify the initial and final states of the system in the contour plots. If the distance between the two states can be represented by a horizontal line, the Rac translocation maybe due to phosphorylation of GDI alone. Prior to stimulus, the cytosolic concentrations of Rac, Cdc42 and GDI are 0.11, 0.15, and 0.39 µM, respectively, resulting in approximately 0.13 µM of free cytosolic GDI. From Section Detailed model, *K_DGDI_* = 1×10^−3^ (white dashed line in Fig. 5A). At time 20 minutes after 20 mM of glucose exposure, there is 40% less Rac bound to GDI, the amount of active Rac in the plasma membrane increases by two fold, and so does the amount of Rac (active plus inactive) at all membranes (including plasma and vesicular) [12], [46], [47]. The interaction between Cdc42 and GDI is unchanged at times 0 and 20 minutes (its redistribution is back to basal levels within five minutes) [12]. There is no active Rac in the granules [50]. We assume that Rac initially has the same binding rate per surface area for plasma and granular membrane (while the off rate depends on whether it is GDP or GTP bound as in Section Detailed model). In summary, prior to stimulus *r0* (plasma plus granular membranes) of Rac is 28%, reaching 57% at 20 minutes of glucose exposure (solid and dashed bold black curves in Fig. 5A). The total surface area of the granules is approximately 3600 µm^2^, the surface area of the plasma membrane is 500 µm^2^, and the cytosolic volume (not including the volume of the 10000 granules) is 850 µm^3^ [48], [49].

Now that the contours for the initial and final states have been identified, it is necessary to identify either *K_Dm_* or *K_DGDI_* for each state. The former depends on GEF/GAP activity and effector concentrations. We choose to identify the latter. Two unknowns are critical: a) the fold decrease in affinity between Rac and sGDI versus Rac and GDI, and b) the initial amount of sGDI. We address the problem by covering a wide range of potential values for both unknowns.

In Fig. 5 we contrast the analysis for the β-cell (surface to volume 4.8/µm, Fig. 5C), and a cell with surface to volume 0.524/µm, as pertains to fibroblasts (Fig. 5B); Fig. 5B also corresponds to the case if Rac did not bind granules in β-cell (plasma membrane surface to volume ∼0.58/µm). The limiting values of *K_Dm_* with total membrane fraction of Rac being either GDP or GTP bound are represented by the horizontal white lines (Fig. 5A–C). While the lower line can be shifted to lower values of *K_Dm_* due to effector binding, the upper is a constraint of the system. Phosphorylated GDI (sGDI) has decreased affinity for Rac [53]. Therefore we address the potential impact of an increase in dissociation constant of 5, 10 and 1000 (or 100 in Fig. 5B) fold (solid black, dashed red, and bold green arrows respectively) for different initial conditions for the fraction of sGDI (different *K_DGDI_*). We solve Eqs.(S21) and (S22) in Text S1 for *K_DGDI_* at times 0 and 20 minutes, given the cytosolic concentrations of Rac, GDI and the 70% increase in sGDI [12]. Given *r0_f_* and *K_DGDI_*, the value of *K_Dm_* is extracted. Each arrow represents an effective solution from the states at time 0 to time 20 minutes: black arrowheads consider Cdc42.GDI to be inert (Eq.S21), while white arrowheads allows phosphorylation of GDI bound to Cdc42 (Eq.S22), with initial conditions for free GDI and GDI bound to Rac, 0.13 and 0.11 respectively. Note the gradual counterclockwise rotation of the arrows with increase of initial *K_DGDI_*. Two values of cytosolic Cdc42 bound to GDI were considered: 0.15 µM [12] and 0.25 µM for comparison, which would result in increased total amount of GDI (dotted green arrow Fig. 5C). (The effect of increased Cdc42 concentration for fixed GDI concentration, i.e., smaller free GDI concentration, is addressed in Fig. S6 in Text S1). For small initial concentrations of sGDI, the solution is unperturbed by Cdc42 cytosolic concentration. However, at higher sGDI levels (starting from higher *K_DGDI_*) the effect of the presence of another GDI binding partner with affinity undisturbed by this phosphorylation is noticeable: the arrow rotates counterclockwise. Still, for a cell with large *Sfc/Vol* (Fig. 5C) the rotation is not enough to turn the arrow horizontal, which would represent a shift in *K_DGDI_* only. This means that phosphorylation of GDI by Pak1 is not the sole mechanism responsible for the translocation of Rac in β-cells. The identical fold increase in membrane bound and active Rac rules out the potential increase in GEF/GAP. Therefore, glucose stimulus is most likely to also promote an increase in affinity between Rac and the membrane (decrease *K_Dm_*), as shown in more detail in the dynamic examples in Fig. 5D (and Fig. S7 in Text S1).

In Fig. 5D the decrease in affinity due to sGDI is ten fold. For simplicity, the GDI bound to Cdc42 is disregarded. Three different types of primary responses were considered upon glucose stimulus (to start at 60 seconds): increase in GDI phosphorylation rate pG (cross), combined with the increase in Rac and plasma membrane association rate kon_M_ (star), and increase in Rac and granular membrane association rate kon_Gr_ (circles). These modulatory effects are represented by the coefficients *a*, *b* and *c* respectively (see definitions in Eqs.(S23)–(S26) in Text S1). The dynamic model was run in Virtual Cell [28], and all parameters and rates can be found in the public model Falkenberg_GTPases_Rac_betaCell (www.vcell.org). The membrane association rate is dependent on the amount of active PLD, represented by PLD*. In agreement with Fig. 5C, Fig. 5D shows that in order to reproduce the experimental observations for fold increase in sGDI and membrane fraction of Rac (1.7 and 2 fold, respectively [12], [46], [47]), it is necessary that the affinity between Rac and membrane is also increased. Such increase must occur for both plasma and vesicle membranes.

## Discussion

We developed a systematic methodology for modeling the cycling of the small GTPases between membrane and cytosol, and their interaction with GDI. We show how to derive the role of GDI from measurable experimental data. The parameters *ρ_GDI_* and *ρ_m_* determine whether localized flux is sufficient to generate a sustained concentration gradient. The dependence of the apparent membrane dissociation rate *k_offAp_* on GDI concentration reveals the importance of GDI mediated versus GDI independent GTPase membrane removal.

The role of GDI in GTPase cycling can be analyzed using the loop described by the lumped model (Fig. 2A). Reversibility requires that the cycle has a null net flux at equilibrium (in which case *ρ_GDI_* = *ρ_m_*). Deviations from this detailed balance are expected in the live cell because it is an open thermodynamic system. However, the larger the net flux, the larger the energy loss. Therefore the contour plots in Fig. 2 (and Fig. S4 in Text S1) are expected to be a good reference for GTPase distribution as a function of the parameter *ρ_Eq_* = *ρ_GDI_* = *ρ_m_*. Charts similar to Fig. 2 may be generated for situations when *ρ_GDI_*≠*ρ_m_* using Eqs.(S14) in Text S1, as long as six out of the eight rates in Fig. 2A are known.

An important conclusion of this study is that the role of GDI in the overall mechanism can change for different GTPases and its cellular or experimental context. For example, while interaction with rdi1 seems to be the fastest pathway to extract Cdc42 in yeast, in NIH3T3 cells the GDI acts as a buffer for Rac (*ρ_Eq_* = 0). In addition, for Cdc42, it's been shown that the inactive GTPase has higher affinity for GDI than the active Cdc42 for membrane bound species [23]. Activation of a GEF for Cdc42 would then increase *K_D1_* and decrease *ρ_GDI_*. The expected outcome is the reduced removal rate of GTPase from the membrane and biased net flow towards the counter-clock direction in Fig. 2A.Note that the results for Cdc42 in yeast suggests that it cycles in a different regime than *in vitro* [23]. While the former has the interaction with rdi1 at the membrane as the preferred pathway for membrane removal of Cdc42, the latter reports identical membrane dissociation rates for GDI free or bound Cdc42.

Rac membrane cycling occurs in a different manner. Based on experimental data from NIH3T3 cells [15], we show that the GDI independent mechanism is the dominant term for *k_offAp_* and Rac cycles in the limit *ρ_Eq_*∼0 of the lumped model. Consequently, the detailed model (including nucleotide state and effector binding) may be simplified by neglecting the dashed reactions in Fig. 1A. A parametric search for the remaining rates in the model suggests that the active Rac is removed from the membrane at a lower rate than the inactive Rac. This means that activation of a Rac GEF will increase Rac membrane fraction by decreasing *K_Dm_*. Another consequence is the apparent higher affinity of cytosolic GDI towards membrane bound inactive rather than active Rac. This model is consistent with the more recent observations that decrease of available GDI results in translocation of GTPase to the membrane, and increase in its active fraction [7]. In addition, we show that degradation of Rac would minimize both effects (Fig. 4C–D).

The residues in GTPases and GDI susceptible to phosphorylation downstream of regulatory pathways have been recently reviewed [54]. The long term effect of GDI and GTPases competing for its binding have also been addressed [7]. Undoubtedly, these studies contribute immensely in understanding the qualitative impact of each of these factors on GTPase behavior. The charts of the lumped model allow visualization of the effect of these modifications on GTPase distribution.

In contrast, only a handful of studies focus on the kinetics between GTPases, GDI and the membrane [15], [21], [23], [42]. We show here that additional quantitative information can be extracted from measurable quantities. The analysis from Section GDI and *k_offAp_* provided the basis for the topology of the model in Section Detailed model. It was essential to perform a model reduction to eliminate parameters/reactions with negligible impact on the observables prior to a parametric search. The presence of non-essential model components increases the number of unknowns and promotes the existence of multiple solutions with equivalent scores.

In β-cells, particularly, due to the large surface area of membranes (including granules), phosphorylation of GDI alone is not sufficient to translocate Rac as observed experimentally. Based on the model of Rac membrane cycling developed in the Section Detailed model, and the parametric plots from the Section Example it is possible to infer that the signaling cascade must promote increased affinity between membranes and Rac. By contrast, phosphorylation of GDI in a system of smaller surface to volume ratio would have been sufficient for Rac translocation (Fig. 5B). In other words, the geometry of the system provided a constraint that allows us to confirm the need for an additional feature in the signaling pathway of the β-cell in order to insure proper Rac redistribution.

It is possible that the parameters extracted using NIH3T3 cells do not relate to β-cells, and it remains unknown if Rac cycling in other mammalian cells have similar behavior, due to lack of experimental data. The key point is that surface to volume ratio is a critical parameter for the analysis of GTPase cycling and would certainly need to be considered in comparing β-cells to fibroblasts. However, the Section Example makes it clear how investigators might benefit from experiments that would provide the parameters necessary to repeat the calculations from Sections Detailed model and Example, not only for Rac, but also for Cdc42 and RhoA. If the experimental datapoints fall in a different region of Fig. 1C, the reactions corresponding to the dashed lines in Fig. 1A must be considered, and *ρ_Eq_* will no longer be null. Nevertheless, the parametric search can be performed using more experimental data points in order to determine all the parameters in Fig. 1A. An analysis similar to the one developed in the Section Example can be repeated using a different chart (or a series of charts) from Fig. 2, instead of Fig. 2B.

Another insight emerges from our analysis of the rates associated with the different mechanisms involved in GTPase. Because the kinetics for vesicle trafficking are two orders of magnitude slower than the other mechanisms, it is likely that the stimulated translocation of Rac from the plasma membrane to vesicles via Rab5 or hormones [55], [56] occurs via a mechanism that enhances the affinity between GTPases and the vesicular/endosome membranes. Note that this mechanism is different than removal/delivery of molecules due to scission/fusion of vesicles.

In summary, we reported a systematic manner of studying GTPase membrane cycling. We identify the relevant terms in membrane cycling via analysis of different parametric groups. We provide the equilibrium solution for the membrane fraction of GTPase cycling in a reversible manner (and the equations for the irreversible scenario). We describe the circumstances in which GDI is inert in removing GTPase from the membrane (*ρ_Eq_* = 0), or it either actively removes it (*ρ_GDI_*>*ρ_m_*) or delivers it to the membrane (*ρ_GDI_*<*ρ_m_*). We show how to use the models to extract parameters from experimental data, and apply to the charts of the compact model. Finally, we used measurable quantities to infer which affinities are being regulated downstream of a signaling pathway. Generally, the methodology and models presented here can be applied to circumstances when concentration levels of GTPases or GDI are altered either through experimental manipulation or a disease state.

### Supporting citations

References [57]–[60] appear in the supporting material Text S1.

## Supporting Information

Text S1 **Further results and supporting material.** Figures S1–S7, equations S1–S26, description of the computational methods, tables S1–S4 with further numerical results, and more detailed description of Rac cycling in β-cells.
(PDF)

## References

[1] Etienne-Manneville S, Hall A (2002) Rho GTPases in cell biology. Nature 420: 629–635. 12478284 10.1038/nature01148

[2] Jaffe AB, Hall A (2005) Rho GTPases: biochemistry and biology. Annu Rev Cell Dev Biol 21: 247–269. 16212495 10.1146/annurev.cellbio.21.020604.150721

[3] Bustelo XR, Sauzeau V, Berenjeno IM (2007) GTP-binding proteins of the Rho/Rac family: regulation, effectors and functions in vivo. Bioessays 29: 356–370. 17373658 10.1002/bies.20558 PMC1971132

[4] Zhao L, Wang H, Li J, Liu Y, Ding Y (2008) Overexpression of Rho GDP-dissociation inhibitor alpha is associated with tumor progression and poor prognosis of colorectal cancer. J Proteome Res 7: 3994–4003. 18651761 10.1021/pr800271b

[5] Harding MA, Theodorescu D (2010) RhoGDI signaling provides targets for cancer therapy. Eur J Cancer 46: 1252–1259. 20347589 10.1016/j.ejca.2010.02.025 PMC11207191

[6] DerMardirossian C, Bokoch GM (2005) GDIs: central regulatory molecules in Rho GTPase activation. Trends Cell Biol 15: 356–363. 15921909 10.1016/j.tcb.2005.05.001

[7] Boulter E, Garcia-Mata R, Guilluy C, Dubash A, Rossi G, et al (2010) Regulation of Rho GTPase crosstalk, degradation and activity by RhoGDI1. Nat Cell Biol 12: 477–483. 20400958 10.1038/ncb2049 PMC2866742

[8] Sasaki T, Kato M, Takai Y (1993) Consequences of weak interaction of rho GDI with the GTP-bound forms of rho p21 and rac p21. J Biol Chem 268: 23959–23963. 8226937

[9] Forget MA, Desrosiers RR, Gingras D, Béliveau R (2002) Phosphorylation states of Cdc42 and RhoA regulate their interactions with Rho GDP dissociation inhibitor and their extraction from biological membranes. Biochem J 361: 243–254. 11772396 10.1042/0264-6021:3610243 PMC1222304

[10] Michaelson D, Ali W, Chiu VK, Bergo M, Silletti J, et al (2005) Postprenylation CAAX processing is required for proper localization of Ras but not Rho GTPases. Mol Biol Cell 16: 1606–1616. 15659645 10.1091/mbc.E04-11-0960 PMC1073645

[11] DerMardirossian C, Rocklin G, Seo JY, Bokoch GM (2006) Phosphorylation of RhoGDI by Src regulates Rho GTPase binding and cytosol-membrane cycling. Mol Biol Cell 17: 4760–4768. 16943322 10.1091/mbc.E06-06-0533 PMC1635405

[12] Wang Z, Thurmond DC (2010) Differential phosphorylation of RhoGDI mediates the distinct cycling of Cdc42 and Rac1 to regulate second-phase insulin secretion. J Biol Chem 285: 6186–6197. 20028975 10.1074/jbc.M109.072421 PMC2825414

[13] Wedlich-Soldner R, Wai SC, Schmidt T, Li R (2004) Robust cell polarity is a dynamic state established by coupling transport and GTPase signaling. J Cell Biol 166: 889–900. 15353546 10.1083/jcb.200405061 PMC2172129

[14] Michaelson D, Silletti J, Murphy G, D'Eustachio P, Rush M, et al (2001) Differential localization of Rho GTPases in live cells: regulation by hypervariable regions and RhoGDI binding. J Cell Biol 152: 111–126. 11149925 10.1083/jcb.152.1.111 PMC2193662

[15] Moissoglu K, Slepchenko BM, Meller N, Horwitz AF, Schwartz MA (2006) In vivo dynamics of Rac-membrane interactions. Mol Biol Cell 17: 2770–2779. 16597700 10.1091/mbc.E06-01-0005 PMC1474787

[16] Lin Q, Fuji RN, Yang W, Cerione RA (2003) RhoGDI is required for Cdc42-mediated cellular transformation. Curr Biol 13: 1469–1479. 12956948 10.1016/s0960-9822(03)00613-4

[17] McDonald P, Veluthakal R, Kaur H, Kowluru A (2007) Biologically active lipids promote trafficking and membrane association of Rac1 in insulin-secreting INS 832/13 cells. Am J Physiol Cell Physiol 292: C1216–1220. 17035298 10.1152/ajpcell.00467.2006

[18] Ugolev Y, Berdichevsky Y, Weinbaum C, Pick E (2008) Dissociation of Rac1(GDP).RhoGDI complexes by the cooperative action of anionic liposomes containing phosphatidylinositol 3,4,5-trisphosphate, Rac guanine nucleotide exchange factor, and GTP. J Biol Chem 283: 22257–22271. 18505730 10.1074/jbc.M800734200 PMC2494937

[19] Gandhi PN, Gibson RM, Tong X, Miyoshi J, Takai Y, et al (2004) An activating mutant of Rac1 that fails to interact with Rho GDP-dissociation inhibitor stimulates membrane ruffling in mammalian cells. Biochem J 378: 409–419. 14629200 10.1042/BJ20030979 PMC1223982

[20] Gibson RM, Gandhi PN, Tong X, Miyoshi J, Takai Y, et al (2004) An activating mutant of Cdc42 that fails to interact with Rho GDP-dissociation inhibitor localizes to the plasma membrane and mediates actin reorganization. Exp Cell Res 301: 211–222. 15530857 10.1016/j.yexcr.2004.07.033

[21] Slaughter BD, Das A, Schwartz JW, Rubinstein B, Li R (2009) Dual modes of cdc42 recycling fine-tune polarized morphogenesis. Dev Cell 17: 823–835. 20059952 10.1016/j.devcel.2009.10.022 PMC2805562

[22] Layton AT, Savage NS, Howell AS, Carroll SY, Drubin DG, et al (2011) Modeling vesicle traffic reveals unexpected consequences for Cdc42p-mediated polarity establishment. Curr Biol 21: 184–194. 21277209 10.1016/j.cub.2011.01.012 PMC3052744

[23] Johnson JL, Erickson JW, Cerione RA (2009) New insights into how the Rho guanine nucleotide dissociation inhibitor regulates the interaction of Cdc42 with membranes. J Biol Chem 284: 23860–23871. 19581296 10.1074/jbc.M109.031815 PMC2749158

[24] Jayaram B, Syed I, Kyathanahalli CN, Rhodes CJ, Kowluru A (2011) Arf nucleotide binding site opener [ARNO] promotes sequential activation of Arf6, Cdc42 and Rac1 and insulin secretion in INS 832/13 β-cells and rat islets. Biochem Pharmacol 81: 1016–1027. 21276423 10.1016/j.bcp.2011.01.006 PMC3073812

[25] Blinov ML, Faeder JR, Goldstein B, Hlavacek WS (2004) BioNetGen: software for rule-based modeling of signal transduction based on the interactions of molecular domains. Bioinformatics 20: 3289–3291. 15217809 10.1093/bioinformatics/bth378

[26] Faeder JR, Blinov ML, Hlavacek WS (2009) Rule-based modeling of biochemical systems with BioNetGen. Methods Mol Biol 500: 113–167. 19399430 10.1007/978-1-59745-525-1_5

[27] Wolfram Research I (2010) Mathematica. Version 8.0 ed. Champaign, Illinois: Wolfram Research.

[28] Moraru II, Schaff JC, Slepchenko BM, Blinov ML, Morgan F, et al (2008) Virtual Cell modelling and simulation software environment. IET Syst Biol 2: 352–362. 19045830 10.1049/iet-syb:20080102 PMC2711391

[29] Cowan AE, Moraru II, Schaff JC, Slepchenko BM, Loew LM (2012) Spatial modeling of cell signaling networks. Methods Cell Biol 110: 195–221. 22482950 10.1016/B978-0-12-388403-9.00008-4 PMC3519356

[30] Slepchenko BM, Loew LM (2010) Use of virtual cell in studies of cellular dynamics. Int Rev Cell Mol Biol 283: 1–56. 20801417 10.1016/S1937-6448(10)83001-1 PMC3519358

[31] Alberts B (2002) Molecular biology of the cell. New York: Garland Science. xxxiv, 1548 p.

[32] Savage NS, Layton AT, Lew DJ (2012) Mechanistic mathematical model of polarity in yeast. Mol Biol Cell 23: 1998–2013. 22438587 10.1091/mbc.E11-10-0837 PMC3350562

[33] Alberts B (2002) Molecular biology of the cell.New York : Garland Science. xxxiv, 1463, [1486] p. p.

[34] Van Aelst L, D'Souza-Schorey C (1997) Rho GTPases and signaling networks. Genes Dev 11: 2295–2322. 9308960 10.1101/gad.11.18.2295

[35] Pertz O (2010) Spatio-temporal Rho GTPase signaling - where are we now? J Cell Sci 123: 1841–1850. 20484664 10.1242/jcs.064345

[36] Novak IL, Gao F, Choi YS, Resasco D, Schaff JC, et al (2007) Diffusion on a Curved Surface Coupled to Diffusion in the Volume: Application to Cell Biology. J Comput Phys 226: 1271–1290. 18836520 10.1016/j.jcp.2007.05.025 PMC2346449

[37] Qian H (2005) Cycle kinetics, steady state thermodynamics and motors-a paradigm for living matter physics. J Phys Condens Matter 17: S3783–3794. 21690724 10.1088/0953-8984/17/47/010

[38] Qian H (2006) Open-system nonequilibrium steady state: statistical thermodynamics, fluctuations, and chemical oscillations. J Phys Chem B 110: 15063–15074. 16884217 10.1021/jp061858z

[39] Chen WW, Schoeberl B, Jasper PJ, Niepel M, Nielsen UB, et al (2009) Input-output behavior of ErbB signaling pathways as revealed by a mass action model trained against dynamic data. Mol Syst Biol 5: 239. 19156131 10.1038/msb.2008.74 PMC2644173

[40] Bielek H, Anselmo A, Dermardirossian C (2009) Morphological and proliferative abnormalities in renal mesangial cells lacking RhoGDI. Cell Signal 21: 1974–1983. 19765647 10.1016/j.cellsig.2009.09.008 PMC2903048

[41] Geiszt M, Dagher MC, Molnár G, Havasi A, Faure J, et al (2001) Characterization of membrane-localized and cytosolic Rac-GTPase-activating proteins in human neutrophil granulocytes: contribution to the regulation of NADPH oxidase. Biochem J 355: 851–858. 11311150 10.1042/bj3550851 PMC1221803

[42] Nomanbhoy TK, Erickson JW, Cerione RA (1999) Kinetics of Cdc42 membrane extraction by Rho-GDI monitored by real-time fluorescence resonance energy transfer. Biochemistry 38: 1744–1750. 10026253 10.1021/bi982198u

[43] Nomanbhoy TK, Cerione R (1996) Characterization of the interaction between RhoGDI and Cdc42Hs using fluorescence spectroscopy. J Biol Chem 271: 10004–10009. 8626553 10.1074/jbc.271.17.10004

[44] Kowluru A (2010) Small G proteins in islet beta-cell function. Endocr Rev 31: 52–78. 19890090 10.1210/er.2009-0022 PMC2852207

[45] Wang Z, Thurmond DC (2009) Mechanisms of biphasic insulin-granule exocytosis - roles of the cytoskeleton, small GTPases and SNARE proteins. J Cell Sci 122: 893–903. 19295123 10.1242/jcs.034355 PMC2720925

[46] Veluthakal R, Madathilparambil SV, McDonald P, Olson LK, Kowluru A (2009) Regulatory roles for Tiam1, a guanine nucleotide exchange factor for Rac1, in glucose-stimulated insulin secretion in pancreatic beta-cells. Biochem Pharmacol 77: 101–113. 18930714 10.1016/j.bcp.2008.09.021 PMC2605786

[47] Li J, Luo R, Kowluru A, Li G (2004) Novel regulation by Rac1 of glucose- and forskolin-induced insulin secretion in INS-1 beta-cells. Am J Physiol Endocrinol Metab 286: E818–827. 14736704 10.1152/ajpendo.00307.2003

[48] Barg S, Eliasson L, Renström E, Rorsman P (2002) A subset of 50 secretory granules in close contact with L-type Ca2+ channels accounts for first-phase insulin secretion in mouse beta-cells. Diabetes 51 Suppl 1: S74–82. 11815462 10.2337/diabetes.51.2007.s74

[49] Rorsman P, Renström E (2003) Insulin granule dynamics in pancreatic beta cells. Diabetologia 46: 1029–1045. 12879249 10.1007/s00125-003-1153-1

[50] Wang Z, Oh E, Thurmond DC (2007) Glucose-stimulated Cdc42 signaling is essential for the second phase of insulin secretion. J Biol Chem 282: 9536–9546. 17289663 10.1074/jbc.M610553200 PMC2396332

[51] Ma WN, Park SY, Han JS (2010) Role of phospholipase D1 in glucose-induced insulin secretion in pancreatic Beta cells. Exp Mol Med 42: 456–464. 20448441 10.3858/emm.2010.42.6.047 PMC2892599

[52] Hughes WE, Elgundi Z, Huang P, Frohman MA, Biden TJ (2004) Phospholipase D1 regulates secretagogue-stimulated insulin release in pancreatic beta-cells. J Biol Chem 279: 27534–27541. 15087463 10.1074/jbc.M403012200

[53] DerMardirossian C, Schnelzer A, Bokoch GM (2004) Phosphorylation of RhoGDI by Pak1 mediates dissociation of Rac GTPase. Mol Cell 15: 117–127. 15225553 10.1016/j.molcel.2004.05.019

[54] Garcia-Mata R, Boulter E, Burridge K (2011) The ‘invisible hand’: regulation of RHO GTPases by RHOGDIs. Nat Rev Mol Cell Biol 12: 493–504. 21779026 10.1038/nrm3153 PMC3260518

[55] Palamidessi A, Frittoli E, Garré M, Faretta M, Mione M, et al (2008) Endocytic trafficking of Rac is required for the spatial restriction of signaling in cell migration. Cell 134: 135–147. 18614017 10.1016/j.cell.2008.05.034

[56] Huang M, Satchell L, Duhadaway JB, Prendergast GC, Laury-Kleintop LD (2011) RhoB links PDGF signaling to cell migration by coordinating activation and localization of Cdc42 and Rac. J Cell Biochem 112: 1572–1584. 21344485 10.1002/jcb.23069 PMC3079809

[57] White FM (1988) Heat and mass transfer. Reading, Mass.: Addison-Wesley. xviii, 718 p. p.

[58] Miura Y, Kikuchi A, Musha T, Kuroda S, Yaku H, et al (1993) Regulation of morphology by rho p21 and its inhibitory GDP/GTP exchange protein (rho GDI) in Swiss 3T3 cells. J Biol Chem 268: 510–515. 8416955

[59] Jenkins GM, Frohman MA (2005) Phospholipase D: a lipid centric review. Cell Mol Life Sci 62: 2305–2316. 16143829 10.1007/s00018-005-5195-z PMC11139095

[60] MacDonald PE, Rorsman P (2007) The ins and outs of secretion from pancreatic beta-cells: control of single-vesicle exo- and endocytosis. Physiology (Bethesda) 22: 113–121. 17420302 10.1152/physiol.00047.2006

