# Supplementary material for: Computational Analysis of Rho GTPase Cycling
Source: PLoS Comput Biol. 2013 Jan 10;9(1):e1002831. doi: 10.1371/journal.pcbi.1002831 (PMC3542069; doi:10.1371/journal.pcbi.1002831)
Supplement: Text S1 — Further results and supporting material. Figures S1–S7, equations S1–S26, description of the computational methods, tables S1–S4 with further numerical results, and more detailed description of Rac cycling in β-cells. (PDF) [file pcbi.1002831.s001.pdf]

Supporting Material:

SM1: Nomenclature

Effect of localized fluxes:

c^* - non-dimensional concentration, i.e., concentration c normalized by characteristic concentration of the system C_o; $c^* \equiv c/C_o$
D_{iff} – diffusion coefficient of GTPase at the membrane
h – total delivery rate of GTPase (GDI mediated, independent and exocytosis)
h_w – net delivery rate of GTPase within the delivery window (delivery minus removal)
L – characteristic length of the sytem
L_w - characteristic length of delivery window
m – total membrane dissociation rate of GTPase (GDI mediated, independent and endocytosis)
x^* - non-dimensional variable for length, i.e. position x normalized by characteristic length of the system L; $x^* \equiv x/L$

ρ_{del} - non-dimensional parameter; ratio between localized and global delivery of GTPase to membrane; Eq.1
ρ_{rem} - non-dimensional parameter; ratio between removal of GTPase from membrane and its diffusive flux; Eq.2

Apparent dissociation rates, lumped and detailed models:

Eff – concentration of effector proteins.
GDI – concentration of GDI molecules.
k_{i-} - unbinding rate of reaction number i.
k_{i+} - binding rate of reaction number i.
k^*_{i+} - binding rate times concentration (of GDI when i=1, 1L, 3, 3L ; of effector when i=5)
K_{Di} - dissociation parameter, ratio k_{i-}/k_{i+}
K^*_{Di} - non-dimensional dissociation parameter, ratio k_{i-}/k^*_{i+}
K_{DGDI} - non-dimensional dissociation parameter between cytosolic GTPase and GDI; Eq.8
K_{Dm} - non-dimensional dissociation parameter between cytosolic membrane and cytosolic GTPase (which is GDI free); Eq.9
k_{offAp} – apparent dissociation rate between GTPase and membrane; Eqs. 4, 5, 16 and S8
$r0$ – fraction of GTPase at the membrane (number of molecules bound to the membrane divided by total number of molecules in the cell); Eq.13
$r0_f$ – fraction of GTPase at the membrane that is free from GDI (number of molecules bound to the membrane that are free from GDI divided by total number of molecules in the cell); Eq.14
Rho_L – lumped concentration of RhoGTPase (includes GDP and GTP bound, and interaction with effector proteins).
Sfc – membrane surface area
Vol – cytosolic volume

ρ_{GDI} - ratio K_{D3L}/K_{D1L} or K^*_{D3L}/K^*_{D1L}; Eq.11

ρ_m - ratio K_{D4L}/K_{D2L}; Eq. 12
ρ_{Eq} - parameter used in detailed balance only, $\rho_{Eq} = \rho_m = \rho_{GDI}$

$(\ )_m$ – membrane bound species/complexes
$(\ )_c$ – cytosolic species/complexes

Example for pancreatic β-cells:

a – coefficient representing increase in phosphorylation rate of GDI upon glucose stimulus; Eq.S24
b – coefficient representing increase in binding rate between cytosolic Rac and plasma membrane due to active phospholipase D; Eq.S25.
c – coefficient representing increase in binding rate between cytosolic Rac and granular membrane due to active phospholipase D; Eq.S26
kon_M – binding rate between cytosolic Rac and plasma membrane; Eq.S25
kon_{Gr} – binding rate between cytosolic Rac and granular membrane; Eq.S26
PLD_{mi} – phospholipase D1, membrane bound and inactive; Eq.S23.
PLD^* – phospholipase D1, membrane bound active; Eq.S23
pG – GDI phosphorylation rate; Eq. S24
sGDI – concentration of GDI that is serine phosphorylated; Eqs.S21-22
$(\ )_t$ – cytosolic GTPase or complex concentration at time t; Eqs.S21-22

SM2. Effect of localized fluxes:

The effect of localized fluxes can be analyzed in a one dimensional Cartesian coordinate system. While the spatial derivatives vary according to the geometry of the problem, the relevant parameters are the same (1).

Consider the GTPase to be delivered with high rate within a specific window of the plasma membrane. The window area is A_w, and the perimeter corresponding to the interface between the window and the outer region is P_w. The characteristic length of the delivery window L_w is defined:

$$L_w \equiv \frac{A_w}{P_w} \qquad \text{(S1)}$$

Within the window, the net delivery rate (flux from the cytosol to the membrane minus the flux from the membrane to the cytosol) is h_w, with units of molecules/(μm^2 s). This delivery rate includes all physiological mechanisms: exocytosis, endocytosis, GDI mediated and independent membrane cycling. Out of the delivery window the delivery rate is defined as h, also with units of molecules/(μm^2 s), and the removal rate is m, with units 1/sec. The membrane diffusion coefficient for the species of interest is D_{iff}.

The conservation of mass out of the window is written as:

$$\frac{\partial c}{\partial t} = D_{iff}\frac{\partial^2 c}{\partial x^2} + h - m\,c \tag{S2}$$

And define the boundary conditions: at the interface between the window and the outer region (x=0) the flux corresponds to the total delivery rate to the window, while there is no net flux at x=L.

$$h_w L_w = -D_{iff}\left.\frac{\partial c}{\partial x}\right|_{x=0} \qquad 0 = -D_{iff}\left.\frac{\partial c}{\partial x}\right|_{x=L} \tag{S3}$$

We use the non-dimensional concentration $c^* \equiv c/C_o$ and the non-dimensional variable $x^* \equiv x/L$. Based on the boundary condition at the delivery window it is convenient to define $Co \equiv (h_w L_w)/(D_{iff}/L)$ and write the system of equations S2-S3 for steady state:

$$\frac{\partial^2 c^*}{\partial x^{*2}} - \rho_{rem}\,c^* = -\frac{1}{\rho_{del}} \qquad \left.\frac{\partial c^*}{\partial x^*}\right|_{x^*=0} = -1 \qquad \left.\frac{\partial c^*}{\partial x^*}\right|_{x^*=1} = 0 \tag{S4}$$

$$\rho_{del} \equiv \frac{h_w}{h}\frac{L_w}{L}$$

$$\rho_{rem} \equiv \frac{m\,L^2}{D_{iff}}$$

The solution for the ratio between the concentrations at $x^*=0$ and at $x^*=1$ is a function of the two parameters ρ_{del} and ρ_{rem}:

$$\frac{C_w}{C_L} \equiv \frac{c^*|_{x^*=0}}{c^*|_{x^*=1}} = \frac{\rho_{del}\sqrt{\rho_{rem}}\cosh\left(\sqrt{\rho_{rem}}\right) + \sinh\left(\sqrt{\rho_{rem}}\right)}{\rho_{del}\sqrt{\rho_{rem}} + \sinh\left(\sqrt{\rho_{rem}}\right)} \tag{S5}$$

The ratio defined in Eq.S5 determines the effect of a localized flux on concentration gradients. When such ratio is the unity, the concentrations in and out of the window are the same. As the ratio gets larger, a concentration gradient will be noticed, i.e., polarization occurs.

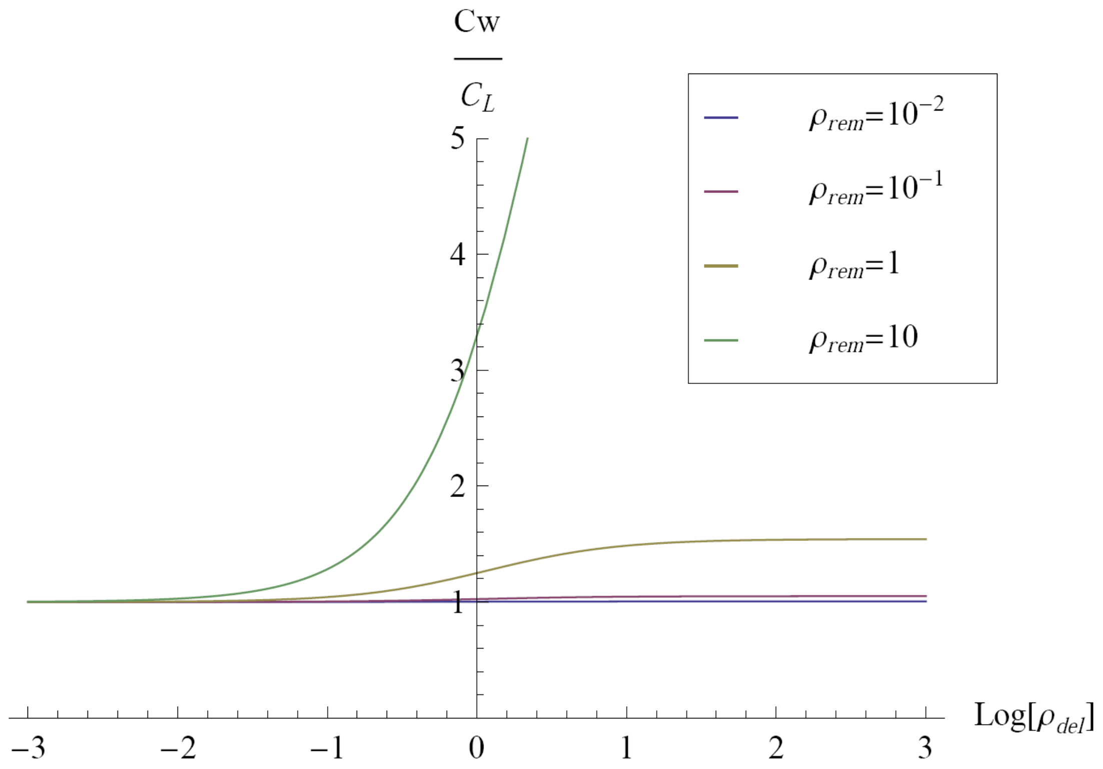

FigureS1. Impact of localized flux (translated into the non-dimensional numbers ρ_{del} and ρ_{rem}) on polarization (gradient in membrane concentration of GTPase). C_w/C_L corresponds to the ratio between concentrations at the delivery window and the opposite boundary.

Figure S1 shows the plots for Eq.S5 as a function of the non-dimensional parameters ρ_{del} and ρ_{rem} for a one dimensional problem on the Cartesian coordinates system. From the analysis becomes clear that localized flux will have negligible impact on membrane concentration profile when ρ_{del}<<1 and ρ_{rem} <<1.

The functional form of the solution will depend on the geometry. However, the solution will always be a function of the parameters ρ_{del} and ρ_{rem}. The generic equation can still be written as:

$$\nabla^2 c^* - \rho_{rem} c^* = -\frac{1}{\rho_{del}} \tag{S6}$$

With the boundary condition for flux normal to the interface with the delivery window:

$$\left.\frac{\partial c^*}{\partial n^*}\right|_w = -1 \tag{S7}$$

The magnitude of the parameter ρ_{rem} determines the contribution of the removal rate and diffusion at the membrane to the spatial gradient. The parameter ρ_{del} represents the ratio between delivery rate at the window (boundary condition at x^*=0) and elsewhere.

As a final note, the removal rate *m* combines the contribution from GDI mediated and independent GTPase removal. The contribution from traffic can be evaluated by the product between the frequency of endocytosis (vesicles per time) times the area of the vesicle divided by the plasma membrane area. If there is a mechanism that increases the concentration of molecules in the vesicle surface in comparison to plasma membrane concentration, the fold increase must also be multiplied.

SM3. Apparent membrane dissociation rate for small GTPases:

Below we describe a dimensional analysis of the apparent membrane dissociation rate (k_{offAp}) of small GTPases due GDI mediated and independent membrane cycling.

For simplicity, the analysis is developed regardless of the nucleotide state. Figure S2a shows spontaneous and GDI mediated membrane dissociation. Figure S2b shows only the reactions for GDI independent membrane cycling of GTPases. Rates with subscript "+" represent binding (to GDI, effector proteins or membrane).

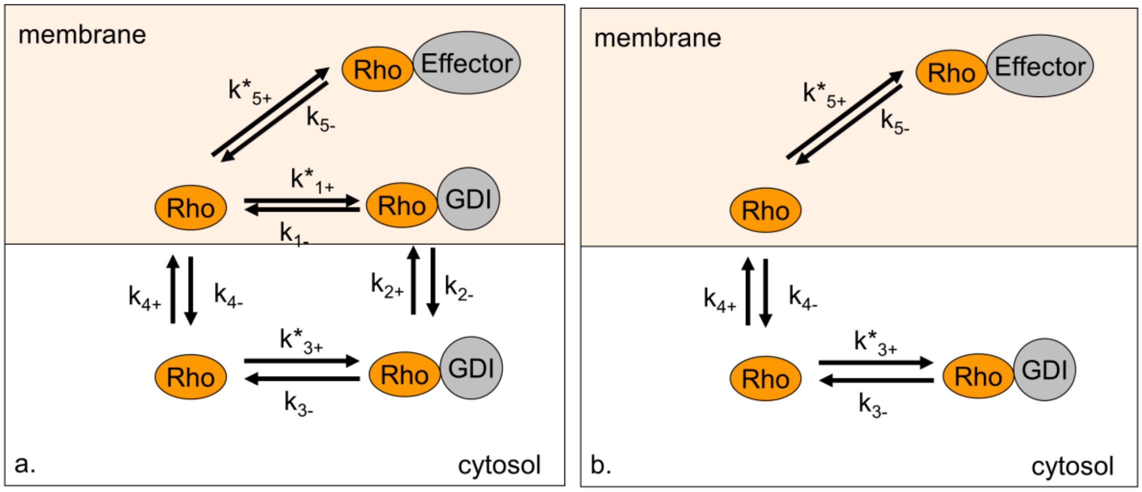

FigureS2. Membrane cycling of RhoGTPases: a. GTPase cycle includes GDI dependent and independent trafficking to and from the membrane; b. GDI independent membrane dissociation of GTPases, without binding between GTPase and GDI while at the membrane. k^*_{1+} and k^*_{3+} correspond to k_{1+} and k_{3+} times free GDI concentration respectively; k^*_{5+} corresponds to k_{5+} times concentration of effector proteins.

The apparent dissociation rate k_{offAp} corresponds to:

$$k_{offAp} = \frac{k_{2-}(Rho \cdot GDI)_m + k_{4-}(Rho)_m}{(Rho + Rho \cdot Eff + Rho \cdot GDI)_m} \tag{S8}$$

Where *Rho* represents the GTPase, a dot identifies two proteins in the same complex, *Eff* represents effector proteins and the subscript *m* represents membrane bound species.

For simplicity, we define the equilibrium dissociation constants:

$$K_{Di} = \frac{k_{i-}}{k_{i+}} \quad , i = \{1, 2, 3, 4, 5\} \tag{S9}$$

The apparent dissociation rate can be split in two terms:

$$k_{offAp} = \frac{k_{2-}(Rho \cdot GDI)_m}{(Rho + Rho \cdot Eff + Rho \cdot GDI)_m} + \frac{k_{4-}(Rho)_m}{(Rho + Rho \cdot Eff + Rho \cdot GDI)_m} \tag{S10}$$

In equilibrium conditions:

$$k_{offAp} = \frac{k_{2-}}{1 + \frac{K_{D1}}{GDI}\left(\frac{Eff}{K_{D5}} + 1\right)} + \frac{k_{4-}}{1 + \frac{GDI}{K_{D1}} + \frac{Eff}{K_{D5}}} \tag{S11}$$

Figure S3 shows plots of k_{offAp} as a function of the ratio between concentration of free GDI and K_{D1} for several values of concentration of effector protein divided by K_{D5}. The first plot considers the GDI independent dissociation rate to be equal to the GDI mediated, while the two other plots consider $k_{4-} < k_{2-}$. The dashed line shows the maximum k_{offAp} in absence of GDI and effector proteins.

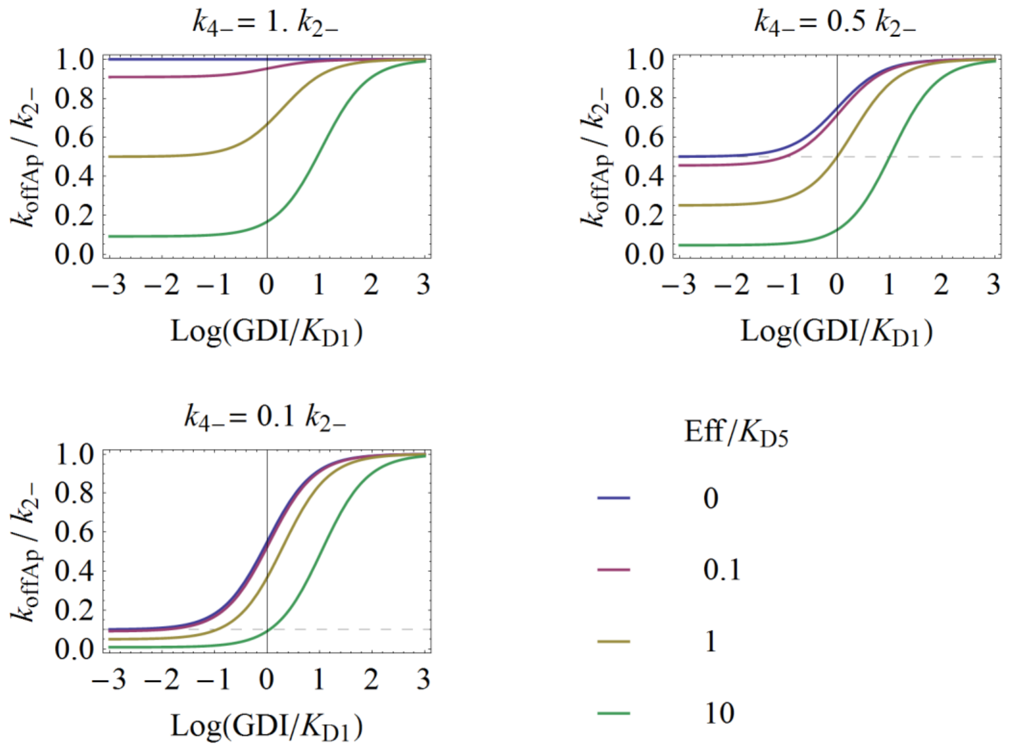

FigureS3. Apparent membrane dissociation rate corresponding to model in FigS2a, normalized by k_{2-}. The concentrations *GDI* and *Eff* correspond to free molecules only. The ratio between k_{4-} and k_{2-} is indicated on the top of each plot.

The independence of k_{offAp} on GDI concentration occurs in two limiting regimes:

$$k_{offAp} \to k_{2-}\ , \qquad for\ \frac{GDI}{K_{D1}} \to \infty$$
$$k_{offAp} \to \frac{k_{4-}}{1+\frac{Eff}{K_{D5}}}\ , \qquad for\ \frac{GDI}{K_{D1}} \to 0 \qquad \text{(S12)}$$

Note that as $GDI \to \infty$ the dissociation rate reaches its maximum. This means that most of GTPase at the membrane is GDI bound. In contrast, as $K_{D1} >> GDI$, no GDI is bound to the GTPase at the membrane.

Transfection of RFP-GDI in cultured NIH3T3 cells (2) showed that the k_{offAp} of GFP-Rac does not depend on GDI expression levels. This observation was reproduced when expressing either GFP-wtRac or its constitutively active form GFP-G12VRac. The RFP-GDI was expressed at different levels, up to several folds the concentration of endogenous GDI.

The k_{offAp} for GFP-wtRac is a linear combination of the k_{offAp} for its GDP and GTP fractions. Because the dissociation rate for the GTP form is independent of the GDI expression level and the k_{offAp} is monotonic with GDI/K_{D1}, the contribution from the GDP form must also be independent of GDI.

This means that Rac cycling (active and inactive) in these cells corresponds to either limiting condition of Eq.S12. Because no GDI was detected at the membrane, it is reasonable to assume that $K_{D1} >> GDI$.

From the detailed balance at equilibrium:

$$\frac{1}{K_{D1}^{*}} \equiv \frac{GDI}{K_{D1}} = \frac{(Rho \cdot GDI)_m}{(Rho)_m}$$
$$\frac{1}{K_{D2}} = \frac{(Rho \cdot GDI)_m}{(Rho \cdot GDI)_c} \qquad \text{(S13)}$$

Where K^{*}_{D1} represents the non-dimensional dissociation constant for the membrane reaction of index 1, and the subscript *c* represents membrane bound species. Eqs.S12-S13 and the absence of detectable membrane bound GDI show that for Rac in NIH3T3 cells $K_{D1}^{*} >> 1$ and $K_{D2} >> 1$.

Based on the experimental observations for Rac in NIH3T3 cells, we propose that the reactions of indexes 1 and 2 can be neglected and the system can be described by the model in Fig.S2b. It is translated into the simplified model of Fig.1d, which accounts for the solid arrows only. In order to verify the proposed model, we searched for a parametric set that would be able to reproduce the experimental observations under several experimental conditions, as described in Section Detailed model.

SM4. Steady State equations for the lumped model (Fig.1c):

$$
\begin{aligned}
&-\left(k^*_{1L+}+k_{4L-}\right)\left(Rho_L\right)_m+k_{1L-}\left(Rho_L.GDI_m\right)+k_{4L+}\left(Rho_L\right)_c=0\\
&-\left(k_{1L-}+k_{2L-}\right)\left(Rho_L.GDI\right)_m+k^*_{1L+}\left(Rho_L\right)_m+k_{2L+}\left(Rho_L.GDI\right)_c=0\\
&-\left(k_{3L-}+k_{2L+}\right)\left(Rho_L.GDI\right)_c+k^*_{3L+}\left(Rho_L\right)_c+k_{2L-}\left(Rho_L.GDI\right)_m=0\\
&-\left(k^*_{3L+}+k_{4L+}\right)\left(Rho_L\right)_c+k_{3L-}\left(Rho_L.GDI\right)_c+k_{4L-}\left(Rho_L\right)_m=0
\end{aligned}
\qquad \text{(S14)}
$$

The subscripts *m* or *c* represents membrane or cytosolic species, respectively.
It is convenient to substitute the numerical indexes by their explicit meanings:

$$
\begin{aligned}
K^*_{D1L}&=\frac{K_{DGDI}}{\alpha_{GDI}}\\
K_{D2L}&=\frac{K_{Dm}}{\alpha_m}\\
K^*_{D3L}&=K_{DGDI}\\
K_{D4L}&=K_{Dm}
\end{aligned}
\qquad \text{(S15)}
$$

The equations for the fraction of GTPase at the membrane *r0*, and the fraction of GTPase at the membrane that is free from GDI, $r0_f$, are in the main text (Eqs. 8-9). Both are valid under detailed balance only. Equations 8-9 can be rewritten as a function of the parameters governing GDI mediated cycling:

$$
r0=\frac{\left(Rho_L\right)_m+\left(Rho_L.GDI\right)_m}{\left(Rho_L\right)_m+\left(Rho_L\right)_c+\left(Rho_L.GDI\right)_c}=\frac{K^*_{D1L}+1}{1+K^*_{D1L}+K_{D2L}+\rho_{Eq}\,K^*_{D1L}K_{D2L}} \qquad \text{(S16)}
$$

$$
r0_f=\frac{\left(Rho_L\right)_m}{\left(Rho_L\right)_m+\left(Rho_L\right)_c+\left(Rho_L.GDI\right)_c}=\frac{K^*_{D1L}}{1+K^*_{D1L}+K_{D2L}+\rho_{Eq}\,K^*_{D1L}K_{D2L}} \qquad \text{(S17)}
$$

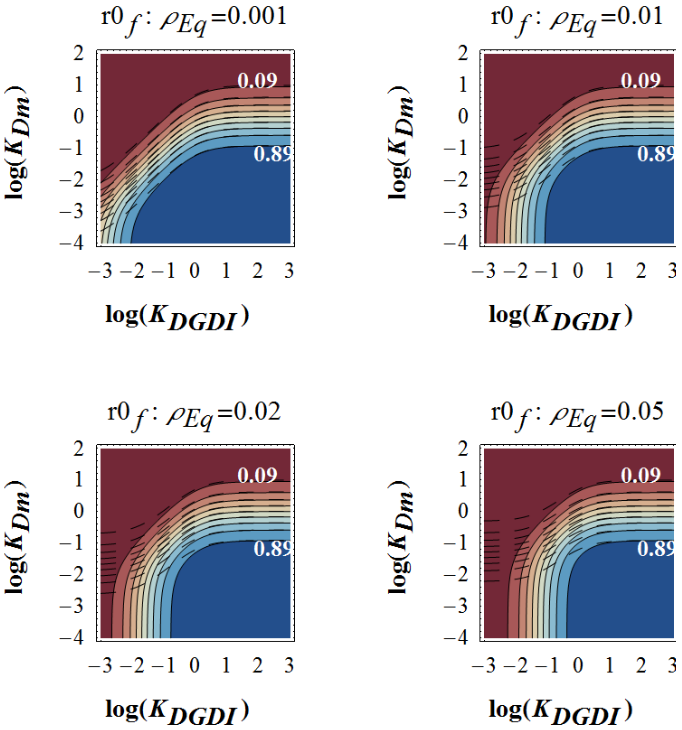

Figure S4. Contour plots representing Eq.10 for ρ_{Eq} ranging from 0.01 to 0.05. The upper contour corresponds to 9% fraction at the membrane, while the lowest line represents 89%. Each pair of neighboring lines is 10% apart in membrane fraction. The total fraction of GTPase at the membrane *r0* is represented by the dashed lines.

SM5. Correlation between reactions described in Fig.1A and Fig.2A:

Note that the nucleotide binding is explicit for variables used in Fig.1A (right hand side of equations), and implicit on variables of Fig.2A (left hand side). Subscripts *m* and *c* stand for membrane bound and cytosolic species, respectively.

$$
\begin{aligned}
(Rho_L)_m &= (RhoGTP.Eff + RhoGTP + RhoGDP)_m \\
(Rho_L.GDI)_m &= (RhoGDP.GDI + RhoGTP.GDI)_m \\
(Rho_L)_c &= (RhoGTP + RhoGDP)_c \\
(Rho_L.GDI)_c &= (RhoGDP.GDI + RhoGTP.GDI)_c
\end{aligned}
$$

$$
\begin{aligned}
k_{1L+}^{*}(Rho_L)_m &= R_{1+,T}^{*}\ (RhoGTP)_m + R_{1+,D}^{*}\ (RhoGDP)_m \\
k_{1L-}(Rho_L.GDI)_m &= R_{1-,T}\ (RhoGTP.GDI)_m + R_{1-,D}\ (RhoGDP.GDI)_m \\
k_{2L+}(Rho_L.GDI)_c &= R_{2+,T}\ (RhoGTP.GDI)_c + R_{2+,D}\ (RhoGDP.GDI)_c \\
k_{2L-}(Rho_L.GDI)_m &= R_{2-,T}\ (RhoGTP.GDI)_m + R_{2-,D}\ (RhoGDP.GDI)_m \\
k_{3L+}^{*}(Rho_L)_c &= R_{3+,T}^{*}\ (RhoGTP)_c + R_{3+,D}^{*}\ (RhoGDP)_c \\
k_{3L-}(Rho_L.GDI)_c &= R_{3-,T}\ (RhoGTP.GDI)_c + R_{3-,D}\ (RhoGDP.GDI)_c \\
k_{4L+}(Rho_L)_c &= R_{4+,T}\ (RhoGTP)_c + R_{4+,D}\ (RhoGDP)_c \\
k_{4L-}(Rho_L)_m &= R_{4-,T}\ (RhoGTP)_m + R_{4-,D}\ (RhoGDP)_m
\end{aligned}
$$

$$
\begin{aligned}
k_{1L+}^{*} &= k_{1L+}\, GDI \\
k_{3L+}^{*} &= k_{3L+}\, GDI \\
R_{1+,T}^{*} &= R_{1+,T}\, GDI \\
R_{3+,T}^{*} &= R_{3+,T}\, GDI \\
R_{1+,D}^{*} &= R_{1+,D}\, GDI \\
R_{1+,D}^{*} &= R_{1+,D}\, GDI
\end{aligned}
\qquad \text{(S18)}
$$

Note that the reaction rates with superscript * are all function of the GDI concentration.

SM6. Equations for detailed model:

$$\frac{d\,(RhoGTP)_m}{dt} = -(R_{0-} + R^*_{1+,T} + R_{4-,T} + R^*_{5+,T})(RhoGTP)_m + R_{0+}(RhoGDP)_m + R_{1-,T}(RhoGTP{\cdot}GDI)_m + R_{4+,T}(RhoGTP)_c + R_{5-,T}(Rho\,GTP{\cdot}Eff\,)_m$$

$$\frac{d\,(RhoGTP\cdot GDI)_m}{dt} = -(R_{1-,T} + R_{2-,T})(Rho\,GTP{\cdot}GDI)_m + R^*_{1+,T}(Rho\,{\cdot}GTP)_m + R_{2+,T}(RhoGTP{\cdot}GDI)_c$$

$$\frac{d\,(RhoGTP\cdot GDI)_c}{dt} = -(R_{2+,T} + R_{3-,T})(RhoGTP{\cdot}GDI)_c + R_{2-,T}(RhoGTP{\cdot}GDI)_m + R^*_{3+,T}(RhoGTP)_c$$

$$\frac{d\,(RhoGTP)_c}{dt} = -(R_{6-} + R^*_{3+,T} + R_{4+,T})(Rho\,GTP)_c + R_{3-,T}(RhoGTP{\cdot}GDI)_c + R_{4-,T}(RhoGTP)_m$$

$$\frac{d\,(RhoGDP)_m}{dt} = -(R_{0+} + R^*_{1+,D} + R_{4-,D})(RhoGDP)_m + R_{0-}(Rho\,GTP)_m + R_{1-,D}(RhoGDP{\cdot}GDI)_m + R_{4+,D}(RhoGDP)_c$$

$$\frac{d\,(RhoGDP\cdot GDI)_m}{dt} = -(R_{1-,D} + R_{2-,D})(RhoGDP{\cdot}GDI)_m + R^*_{1+,D}(RhoGDP)_m + R_{2+,D}(RhoGDP{\cdot}GDI)_c$$

$$\frac{d\,(RhoGDP\cdot GDI)_c}{dt} = -(R_{2+,D} + R_{3-,D})(RhoGDP{\cdot}GDI)_c + R_{2-,D}(RhoGDP{\cdot}GDI)_m + R^*_{3+,D}(RhoGDP)_c$$

$$\frac{d\,(RhoGDP)_c}{dt} = -(R_{6-} + R^*_{3+,D} + R_{4+,D})(RhoGDP)_c + R_{3-,D}(RhoGDP{\cdot}GDI)_c + R_{4-,D}(RhoGDP)_m$$

$$\frac{d\,(RhoGTP\cdot Eff\,)_m}{dt} = -R_{5-,T}(RhoGTP{\cdot}Eff\,)_m + R^*_{5+,T}(RhoGTP)_m$$

$$R^*_{1+,T} = R_{1+,T}\ GDI \qquad \text{(S19)}$$
$$R^*_{1+,D} = R_{1+,D}\ GDI$$
$$R^*_{3+,T} = R_{3+,T}\ GDI$$
$$R^*_{3+,D} = R_{3+,D}\ GDI$$
$$R^*_{5+,T} = R_{5+,T}\ Eff$$

$$GDI_{Tot} = GDI + (RhoGTP{\cdot}GDI)_m + (RhoGDP{\cdot}GDI)_m + (RhoGTP{\cdot}GDI)_c + (RhoGDP{\cdot}GDI)_c$$
$$Eff_{Tot} = Eff + (RhoGTP{\cdot}Eff\,)_m$$

SM.7 Model simplifications used in Section Detailed model:

In order to compare the model with experimental procedures that use transfected GFP-Rac and its constitutively active form GFP-G12VRac, we take into account the endogenous and transfected species. The same reactions defined for endogenous Rac are also considered for transfected GFP-Rac. The constitutively active form is not subject to deactivation by GAP or any reaction involving Rho-GDP in Fig.1A. All reaction rates for both GFP forms are taken to be identical to their respective rates for endogenous Rac.

The model is constrained with the following experimentally derived observations:
a) The membrane dissociation rate of Rac-GDP is higher than the Rac-GTP. b) Both active and inactive Rac bind to the membrane at the same rate. c) The cytosolic GAP activity is 4 times higher than at the membrane. This value corresponds to the maximum increase in binding rate due to diffusion of 2 molecules in the cytosol in comparison to one of them being fixed at the membrane. It is also consistent with experimental observations(3). d) There is negligible Rac-GDI at the membrane. e) GFP-G12V has the same kinetics as GFP-wtRac-GTP in terms of effector and membrane binding/unbinding. f) The total amount of endogenous GDI is three times the GFP-Rac. g) For GDI knockdown experiments, the total amount of Rac is assumed to be one half that in other experiments. This assumption is based on imunoblot experiments showing reduced total Rac in comparison to control experiments. Similar observation was reported for GDI-/- renal mesangial cells (4). h) Rac bound to effectors dissociate from membrane at negligible rate. The data for total active GFP-G12VRac is also used as a constraint since the expression of either GFP-wtRac or GFP-G12VRac were expected to be similar in all experiments. The total amount of GFP-G12VRac follows the experimental data, while the amount of GFP-wtRac is expected to be the average of what was reported for GFP-G12VRac.

The reaction rules are conveniently defined using BioNetGen (5, 6), with molecules and complexes comprising Rac with a binding site for nucleotides (GDP and GTP), a binding site for GDI or effector (exclusive binding) and cell localizations (membrane and cytosol); the BioNetGen code is provided in Fig.S5. Note that based on the experimentally derived evidence, the reactions with subscript indexes 1 and 2 in Fig. 1 are neglected. The system of equations generated by BioNetGen is exported to Matlab (7), where the optimization function 'fmincon' with the interior-point algorithm is used to optimize for parametric values of: a) membrane dissociation rate of active Rac; b) membrane dissociation rate of inactive Rac; c) basal level of GEF activity; d) increase in GEF activity due to Tiam1 co-transfection; e) amount of GFP-Rac; f) ratio between endogenous and GFP Rac; g) the dissociation constant between cytosolic GDP bound Rac and GDI; h) ratio between the latter value in comparison to GTP bound Rac; i) fold increase in GDI concentration for GDI overexpression experiments; j) fraction decrease in GDI knockdown experiments; and k) association rate between Rac and the membrane.

The BNG code is in the end of the Supporting Material.
The bngl model was run using VCell using the menu Tools/Launch BioNetGen.
The optimized values for the parameters are already updated in the file.

```
# gef, gap
rac(s1,s2!2,loc~Memb).GDP(s2!2,loc~Memb)            <->  rac(s1,s2!2,loc~Memb).GTP(s2!2,loc~Memb)                       gef*pmp0, gap*pmp0
rac(s1,s2!2,loc~Cyt).GTP(s2!2,loc~Cyt)              ->   rac(s1,s2!2,loc~Cyt).GDP(s2!2,loc~Cyt)                         gap*4
#binding to effector
rac(s1,s2!2,loc~Memb).GTP(s2!2,loc~Memb)+eff(s1,loc~Memb)  <->  rac(s1!1,s2!2,loc~Memb).GTP(s2!2,loc~Memb).eff(s1!1,loc~Memb)  konE*pmp0*pmp0/pmp, koffE*pmp0
G12V(s1,loc~Memb)+eff(s1,loc~Memb)                         <->  G12V(s1!1,loc~Memb).eff(s1!1,loc~Memb)                          konE*pmp0*pmp0/pmp, koffE*pmp0

#translocation between membrane and cytosol
rac(s1,s2!2,loc~Memb).GDP(s2!2,loc~Memb)            <->  rac(s1,s2!2,loc~Cyt).GDP(s2!2,loc~Cyt)                         koffMD*pmp0,konM*pmp0
rac(s1,s2!2,loc~Memb).GTP(s2!2,loc~Memb)            <->  rac(s1,s2!2,loc~Cyt).GTP(s2!2,loc~Cyt)                         koffMT*pmp0,konM*pmp0
G12V(s1,loc~Memb)                                   <->  G12V(s1,loc~Cyt)                                               koffMT*pmp0,konM*pmp0

# binding to GDI
rac(s1,s2!2,loc~Cyt).GDP(s2!2,loc~Cyt)+GDI(s1,loc~Cyt)  <->  rac(s1!1,s2!2,loc~Cyt).GDP(s2!2,loc~Cyt).GDI(s1!1,loc~Cyt)   konDG, koffDG
rac(s1,s2!2,loc~Cyt).GTP(s2!2,loc~Cyt)+GDI(s1,loc~Cyt)  <->  rac(s1!1,s2!2,loc~Cyt).GTP(s2!2,loc~Cyt).GDI(s1!1,loc~Cyt)   konTG, koffTG
G12V(s1,loc~Cyt)+GDI(s1,loc~Cyt)                        <->  G12V(s1!1,loc~Cyt).GDI(s1!1,loc~Cyt)                          konTG, koffTG
```

Figure S5. Reaction rules defined in BioNetGen for the Rac model. Since both endogenous Rac and GFP-wtRac are subject to the same reactions, they are defined as a single variable, while the active form GFP-G12VRac is defined as a second variable. Following the experimentally derived postulates for Rac, reactions with subscript indexes 1 and 2 in Fig.1A are neglected.

SM8. Parametric Search:

Parametric search

Table S1 show the list of parameters that were optimized using the Matlab function “fmincon”, with the upper and lower boundaries for each parameter as shown. The optimization is performed for total effector concentrations of 0.05, 0.1, 0.3, 0.5 and 1 μM to test the sensitivity of the other model parameters to this variable. To assure that local false minima were avoided, the optimizations were repeated with a range of initial guesses. The endogenous and transfected concentrations for Rac and GDI in NIH3T3 cells were not reported (2). The authors do report that the concentration of endogenous GDI is about three fold the concentration of expressed GFP-Rac. The latter is approximately the concentration of endogenous Rac (personal communications). Endogenous GDI in Swiss 3T3 cells were reported to be about 0.1 μM (8). The initial guess values for the expression of GFP-Rac where 0.001, 0.005, 0.01, 0.025 and 0.04 μM. The initial guess values for fold increase in basal GEF activity due to tiam1 cotransfection of 2, 5 and 10. The optimization is performed aiming to minimize the total error between numerical simulations and experimental data. The total error is a weighed sum of individual errors of three properties ($r0$, $k_{off\,Ap}$ and total active GFP-Rac in the cell) for the 8 different experimental conditions described above (there was data available for 20 experimental values, used to fit 11 parameters). Each individual error is evaluated as the normalized difference between probability density functions of the numerical result and experimental data. The normal distribution is assumed for all experimental data.

The final score Sc is defined as a function of the normalized probability density function (pdf) of the numerical solution “M” relative to the experimental data “exp”.

$$Sc = \frac{\sum_j \sum_i \left(w(i,j) \left(1 - \frac{pdf(M_{i,j}, s_{i,j})}{pdf(\exp_{i,j}, s_{i,j})} \right) \right)}{\sum_j \sum_i \left(w(i,j) \right)} \tag{S20}$$

$$i = \{r0, koffAp, activeRac\},$$

$$j = \{wt, wt + tiam, wt + GDI, wt - GDI, G12V, G12V + tiam, G12V + GDI, G12V - GDI\}$$

$$w(i,j) = \begin{Bmatrix} 2 & 2 & 2 & 2 & 2 & 2 & 2 & 2 \\ 2 & 2 & 2 & 2 & 2 & 0 & 2 & 0 \\ 0 & 2 & 1 & 1 & 1 & 1 & 1 & 0 \end{Bmatrix}$$

The values for the weigh function *w(i,j)* are null in the absence of experimental data. Lower weigh values were chosen for the data of active Rac. Since the latter experimental values are obtained by a ratio (amount of active Rac in the current experiment divided by the value obtained when expressing wt-Rac alone), their values may be less reliable than the measurements for r0 and k_{offAp}. We noticed significant improvement on the parametric optimization by assigning *w*(wt+tiam,activeRac)=2.

TableS1: Parametric search allowing different membrane dissociation rate for RacGDP and Rac GTP.

Parameter number and correspondence in the model		Lower bound	Upper bound	Initial guess	Final value: Fig 3
P1	$R_{4-,D}$	0	1	0.05	0.149611
P2	$R_{4-,T}$	0	.02	0.004	0.011128
P3	R_{0+}	0	20	2	18.79167
P4	$(R_{0+,wt+tiam})/(P3 \times tm)$	0	100	1*	1.628607
P5	(GFP-Rac)/g12	0	50	1*	4.616598
P6	$R_{3-,D}$	0	1	0.001	0.000130289
P7	GDI_{+GDI}/GDI_c	1	5	4	2.845271
P8	GDI_{-GDI}/GDI_c	0	0.5	0.1	1.741033
P9	$(Rac_{GFP})/(Rac_c)$	0.5	2	1	1.741033
P10	P6/ $R_{3-,T}$	0.01	1	1	0.998463
P11	R_{4+}/P1	1	100	20	18.99576
**Initial guess***		**List of values**			
tm	Used in P4	2, 5, 10			2
g12	Used in P5	0.001, 0.005, 0.01, 0.025, 0.04			0.01
Eff	Effector concentration, μM	1, 0.75, 0.5, 0.3, 0.1			0.5

$R_{0+,wt+tiam}$= rate R_{0+} for experimental conditions wt+tiam and G12V+tiam;
Rac_{GFP} = concentration of transfected Rac;
Rac_c= concentration of endogenous Rac;
GDI_c = concentration of endogenous GDI;
GDI_{+GDI} = concentration of GDI after cotransfection;
GDI_{-GDI} = concentration of GDI after knockdown.
* The parametric search was performed for 75 combinations of initial guess, by modifying the initial guesses for P4, P5 and effector concentration using the listed values for "tm" and "g12".

Table S2 shows the top 5 parametric sets performed with the same initial conditions as listed in Table S1. The score of the parametric group ranges from 0 to 1, with 0 corresponding to a perfect match to the experimental data presented in Fig. 3.

Table S2: Score and parameter values of top 5 results. P6*=P6 x 1e3.

Sc.	P1	P2	P3	P4	P5	P6*	P7	P8	P9	P10	P11	tm	g12	Eff
0.1606	0.1496	0.0111	18.7917	1.6286	4.6166	0.1303	2.8453	0.3689	1.7410	0.9985	18.9958	2	.01	.5
0.1761	0.1350	0.0127	19.5761	1.6715	2.5237	0.3653	3.4152	0.3811	1.5038	0.9057	21.0061	2	.04	.5
0.1916	0.2004	0.0109	19.9236	1.6382	41.7886	1.3789	4.9125	0.3916	1.3417	1.0000	20.7261	2	.01	1
0.2248	0.0911	0.0145	7.9194	1.5310	10.8562	1.3307	3.3795	0.4045	1.9863	0.6779	19.8197	2	.025	.75
0.2489	0.0624	0.0102	4.4700	1.0106	1.0359	0.0142	4.1006	0.3515	0.8013	0.9386	16.5448	2	.001	.3

TableS3 shows the values of concentrations and kinetic rates resulting from the optimized parametric set. Numbers in bold represent results from the parametric search.

TableS3: Concentrations and rates corresponding to best parametric set.

**Concentrations**			**units**	**comments**
GDI, total	0.149		μM	GFP_Rac x 3
Rac, total	GFP: **0.046**; endogenous: **0.080**		μM	
Effector proteins, total	0.5		μM	
**Rates**	**D**	**T**		
R_{0+}	**18.79**		1/s	
R_{0-}	10		1/s	
R_{3+}	1	1	1/(μM s)	
R_{3-}	**1.3029e-4**	**1.3048e-4**	1/s	$R_{3-,D}$ / $R_{3-,T}$ =**0.9984**
R_{4+}	**2.842**		1/s	
R_{4-}	**0.1496**	**0.0111**	1/s	
R_{5-}	0.1		1/s	
R_{5+}	0.1		1/(μM s)	
R_{6-}	40		1/s	
**Modifications due to co-transfections or knockdown**				
**+ tiam:** R_{0+}	**61.06**		1/s	
**+ GDI**: GDI, total	**0.423**		μM	GDI x **2.845**
**- GDI**: GDI, total	**0.055**		μM	GDI x **0.369**
**- GDI**: Rac, total	GFP: 0.023; endogenous: 0.040		μM	Rac x 0.5

Same membrane dissociation rate for RacGDP and Rac GTP. Another set of 75 searches were performed with $R_{4\text{-}D}=R_{4\text{-}T}$, as listed in TableS4. The top 5 results for this functional form is poorer than for $R_{4\text{-}D}\neq R_{4\text{-}T}$ (Tables S1 and S2), with Sc ranging from 0.239 to 0.31.

TableS4.

**Parameter number and correspondence in the model**		**Lower bound**	**Upper bound**	**Initial guess**	**Final value: Sc=0.2392**
P1	$R_{4-,D} = R_{4-,T}$	0	1	0.05	0.112070
P2	R_{5-} / R_{5+}	0	10	1	0.641848
P3	R_{0+}	0	20	2	1.295287
P4	$(R_{0+,wt+tiam})/(P3 \times tm)$	0	100	1*	29.17745
P5	(GFP-Rac)/g12	0	50	1*	37.80201
P6	$R_{3-,D}$	0	1	0.001	0.000459542
P7	GDI_{+GDI}/GDI_c	1	5	4	3.994331
P8	GDI_{-GDI}/GDI_c	0	0.5	0.1	0.372222
P9	$(Rac_{GFP})/(Rac_c)$	0.5	2	1	1.963508
P10	$P6/ R_{3-,T}$	0.01	1	1	0.043729
P11	$R_{4+}/P1$	1	100	20	21.72582
**Initial guess***		**List of values**			
tm	Used in P4	2, 5, 10			2
g12	Used in P5	0.001, 0.005, 0.01, 0.025, 0.04			0.01
Eff	Effector concentration, μM	10,1, 0.75, 0.5, 0.3			0.5

$R_{0+,wt+tiam}$= rate R_{0+} for experimental conditions wt+tiam and G12V+tiam;
Rac_{GFP} = concentration of transfected Rac;
Rac_c= concentration of endogenous Rac;
GDI_c = concentration of endogenous GDI;
GDI_{+GDI} = concentration of GDI after cotransfection;
GDI_{-GDI} = concentration of GDI after knockdown.
* The parametric search was performed for 75 combinations of initial guess, by modifying the initial guesses for P4, P5 and effector concentration using the listed values for "tm" and "g12".

Table S5: Score and parameter values of top 5 results for search with $R_{4\text{-}D}=R_{4\text{-}T}$. P6*=P6 x 1e3.

**Sc.**	**P1**	**P2**	**P3**	**P4**	**P5**	**P6***	**P7**	**P8**	**P9**	**P10**	**P11**	**tm**	**g12**	**Eff**
0.2392	0.1127	0.6418	1.2952	29.177	37.8020	0.4595	3.9943	0.3722	1.9635	0.4397	21.725	5	0.005	10
0.2520	0.1434	0.6400	2.9319	0.9999	1.04325	0.0055	4.2032	0.4346	0.9008	0.7820	19.655	5	0.001	10
0.2714	0.0600	0.1558	5.3770	5.4784	4.53409	0.8063	3.3359	0.3779	1.6078	0.3794	19.951	2	0.04	0.75
0.3095	0.0504	0.1552	1.5593	1.1710	2.29297	0.9961	4.5531	0.4807	1.9666	0.6073	20.034	10	0.04	0.75
0.3100	0.0386	2.2229	0.0975	1.5913	0.05809	0.0380	4.5291	0.3512	0.6333	0.9483	21.845	5	0.04	10

SM9. Insulin secretion by pancreatic β cell:

The biphasic insulin release from the β-cells of the pancreatic islets involves several modifications on the kinetic parameters for cycling of the small GTPases Cdc42 and Rac between cytosol and membrane, promoting the prolonged glucose stimulated insulin release.

The first stage of secretion consists of fusion of pre docked granules, resulting on a peak of insulin release. The second stage occurs within 10 minutes, requires traffic of the 'storage-granule pool' to the cell surface and results in insulin release at lower yet sustained rate.

Glucose stimulus has been shown to trigger activation of Arf6 (small G-protein ADP ribosylation factor) within one minute (9), followed by transient translocation from cytosol to membrane and stimulation of Cdc42 within three minutes (back to resting levels by 5 minutes) and translocation to the plasma membrane and stimulation of Rac1 after fifteen minutes (10, 11). Inhibition of any of these activation steps results in significant reduction on the activation of the subsequential step(s) and consequently reduced insulin secretion during the second stage.

The connection between activation of Arf6 and Cdc42 is not clear. However, a combination of experiments suggests two contributing mechanisms for the downstream activation of Rac1(9). Upon glucose stimulation, Arf6 has been shown to translocate from cytosol to plasma membrane, where it activates Phospholipase D1 (PLD1) (12). PLD1 is predominantly localized on insulin granules at basal conditions and found at the plasma membrane upon fusion of the insulin granules. Its activation (by Arf6, PKC or RhoGTPases; (13)) results in increased phosphatidic acid (PtdOH), while the inhibition of PtdOH production significantly decreases insulin secretion on first and second stages of insulin secretion (14). Most importantly, PtdOH has been shown to increase Rac membrane fraction (15). This modification occurs over several minutes, since granular fusion on the second stage of insulin secretion is constant over more than thirty minutes.

The second mechanism essential for Rac translocation is the activation of Pak1 by Cdc42, possibly due to GDI serine phosphorylation by Pak1. Depletion of either Cdc42 or Pak1 will completely inhibit Rac activation by glucose, and disrupt the second stage of insulin secretion, reducing it by half (11, 16). Since inhibition of the first mechanism abrogates granule fusion at the first stage already, it is possible that the second mechanism is being prevented altogether. Therefore, it is not clear from the experiments whether there is significant contribution of the modified lipid composition on Rac translocation. However, the observations on the second mechanism show that the first one alone is not enough to promote Rac release from GDI and activation.

The two mechanisms are considered in Section Example, Figs. 5, S6. The arrows on Fig. 5 were obtained solving the system of equations below (Eq. S21 for solid arrowheads and Eq. S22 for white arrowheads). The equations relate experimental data on cytosolic concentrations of GTPases and GDI at times 0 and 20 minutes. Because each point in the plot has the information of membrane fraction $r0$, K_{DGDI} (or K_{D3}) and K_{Dm}, we can use the following protocol: solve Eqs.S21 or S22 for K_{DGDI}, mark in the plot its intersection with $r0$, and we automatically extract the value of K_{Dm}.

Equations for solid arrowheads in Fig. 5:

$$\left(K^*_{D3L}\right)_t = \frac{\left(K_{D3L}\right)_t}{(GDI)_t + (sGDI)_t} = \frac{\left(Rac_L\right)_t}{\left(Rac_L.GDI\right)_t + \left(Rac_L.sGDI\right)_t} \qquad t = \{0, 20\}$$

$$1.3 \times 10^{-4} = \frac{\left(Rac_L\right)_t \ (GDI)_t}{\left(Rac_L.GDI\right)_t}$$

$$coef \times 1.3 \times 10^{-4} = \frac{\left(Rac_L\right)_t \ (sGDI)_t}{\left(Rac_L.sGDI\right)_t}$$

$$\left(Rac_L\right)_0 + \left(Rac_L.GDI\right)_0 + \left(Rac_L.sGDI\right)_0 = 0.11$$

$$\left(Rac_L\right)_{20} + \left(Rac_L.GDI\right)_{20} + \left(Rac_L.sGDI\right)_{20} = 0.0622 \qquad \text{(S21)}$$

$$GDI_{tot} = \left(Rac_L.GDI\right)_t + \left(Rac_L.sGDI\right)_t + (GDI)_t + (sGDI)_t$$

$$GDI_{tot} = 0.13 + 0.11$$

$$(sGDI)_{20} + \left(Rac_L.sGSI\right)_{20} = 1.7\left((sGDI)_0 + \left(Rac_L.sGSI\right)_0\right)$$

Where a pair of bound proteins is represented by a dot, the subscripts *0*, *20* or *t* represent time in minutes, the subscript *tot* represents total amount. Concentrations are in μM. K_{Dc} corresponds to the dissociation constant between cytosolic Rac and GDI, in μM. Phosphorylated GDI is represented by *sGDI,* while unphosphorylated by *GDI*. The fold increase in dissociation constant is represented by *coef*.

Equations for solutions represented by arrows with white arrowheads in Fig. 5:

$$\left(K^*_{D3L}\right)_t = \frac{\left(K_{D3L}\right)_t}{(GDI)_t + (sGDI)_t} = \frac{\left(Rac_L\right)_t}{\left(Rac_L.GDI\right)_t + \left(Rac_L.sGDI\right)_t} \qquad t = \{0, 20\}$$

$$1.3 \times 10^{-4} = \frac{\left(Rac_L\right)_t \ (GDI)_t}{\left(Rac_L.GDI\right)_t}$$

$$coef \times 1.3 \times 10^{-4} = \frac{\left(Rac_L\right)_t \ (sGDI)_t}{\left(Rac_L.sGDI\right)_t}$$

$$\frac{\left(Cdc42_L.GDI\right)_t}{\left(Cdc42_L.sGDI\right)_t} = \frac{(GDI)_t}{(sGDI)_t} \qquad \text{(S22)}$$

$$\left(Cdc42_L.GDI\right)_t + \left(Cdc42_L.sGDI\right)_t = 0.15$$

$$\left(Rac_L\right)_0 + \left(Rac_L.GDI\right)_0 + \left(Rac_L.sGDI\right)_0 = 0.11$$

$$\left(Rac_L\right)_{20} + \left(Rac_L.GDI\right)_{20} + \left(Rac_L.sGDI\right)_{20} = 0.0622$$

$$GDI_{tot} = \left(Rac_L.GDI\right)_t + \left(Rac_L.sGDI\right)_t + \left(Cdc42_L.GDI\right)_t + \left(Cdc42_L.sGDI\right)_t + (GDI)_t + (sGDI)_t$$

$$GDI_{tot} = 0.13 + 0.11 + 0.15$$

$$(sGDI)_{20} + \left(Rac_L.sGSI\right)_{20} + \left(Cdc42_L.sGDI\right)_{20} = 1.7\left((sGDI)_0 + \left(Rac_L.sGSI\right)_0 + \left(Cdc42_L.sGDI\right)_0\right)$$

The horizontal solid lines in Fig. 5a-c correspond to values of K_{D4L} if all membrane bound Rac is GDP bound (upper line) or GTP bound and free from effector (lower bound), using Eq.2. Upon binding to effector proteins, K_{D4L} may be further reduced.

The Eqs. S21 and S22 were obtained under the simplification that the timescales for Rac membrane cycling and granular fusion are of different orders of magnitude. The fusion of granules to the plasma membrane results in the increase of surface area of the cell. Such increase can be estimated by measurement of increase in cell capacitance. Each cell has the surface area 500 μm^2 (17). Since each granule has area of 0.36 μm^2 (18), the maximum release of 200 granules during the first phase secretion (19) would represent a cell surface area increase of 14% over the course of 10 minutes in the absence of any endocytosis. Endocytosis in β-cells occur in two time scales, with 7-60% being fast, by 'kiss-and-run', and the remaining by stimulated conventional endocytosis (20). For simplicity, here we assume that the total endocytosis is able to maintain the cell surface area constant at all times. The significant occurrence of conventional endocytosis is important to justify delivery of granular PLD1 to plasma membrane (14), and decrease K_{Dm}. The delivery of granule bound molecules to the plasma membrane due to fusion rate is on the order of 10^{-4}/s (and so we assume to be endocytosis). Since Rac binds to and leaves the membrane at rates 10^{-2}/s and faster, and Rac granular and membrane concentration are on the same order (16), fusion and endocytosis of individual granules have negligible effect on Rac membrane cycling rates.

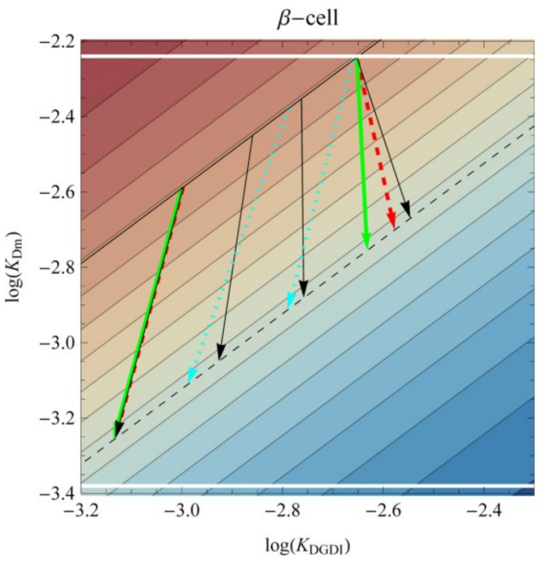

Figure S6. Membrane fraction of Rac before and after increase in serine phosphorylated GDI due to stimulus. Horizontal lines delimit K_{Dm} for minimum and maximum GEF/GAP based on values in Section Detailed model, taking into account the geometry of β-cell line. Arrows represent trajectories that satisfy the 70% increase in serine phosphorylated GDI and 40% decrease in cytosolic Rac. Each color correspond to a different fold increase in dissociation constant between Rac and serine phosporylated GDI in comparison to unphosphorylated GDI (see text). Fold increase: 5, black; 10, red dashed; 1000 green bold and dotted blue arrows. Black, red and green arrows are repeated from Fig.5c. GDI bound to Cdc42 was considered inert and there is 0.13μM of free cytosolic GDI, 0.13 μM of

cytosolic Rac and 0.20 μM of cytosolic Cdc42 at t=0. Blue arrow includes phosphorylation of GDI bound to Cdc42, with 0.08 μM of free GDI, 0.13 μM of cytosolic Rac and 0.20 μM of cytosolic Cdc42 at t=0.

Transient example for pancreatic β-cell.

The purpose of the examples in Figs. 5D and S7 is to further explain Fig. 5C.

The Membrane cycling BNG script was exported to VCell where it was modified to accommodate the parametric changes (BIOMODEL: Falkenberg_GTPases_Rac_betaCell). An analytical expression was added to represent the fusion of granules to the membrane. This expression is based on the measurements of transient insulin release (16, 17).

Upon fusion of granules, PLD1 is transferred from the granules to the membrane in its inactive form PLD_{mi}. At the membrane it becomes activated PLD^*. The activation rate is considered to occur much faster than the traffic. Because the delivery rate is slow, both quantities PLD_{mi} and PLD^* become a function of time t:

$$PLD^*(t) = \frac{PLD_{mi}(t)}{0.1 + PLD_{mi}(t)} \qquad \text{(S23)}$$

The association rate between Rac and the membrane (Eq.7) will be time dependent due to progressive increase in the total amount of activated PLD^*. Because we allow the association rate between cytosolic GTPase to be different towards plasma or granular membrane, k_{4L+} is substituted by kon_M and kon_{Gr}, respectively. We define the GDI phosphorylation rate pG, the Rac and plasma membrane association rate kon_M, and the Rac and granular membrane association rate kon_{Gr}:

$$pG(t) = pG(0)\cdot(1+a) \qquad \text{(S24)}$$
$$kon_M(t) = kon_M(0)\cdot\left(1+b\ PLD^*(t)\right) \qquad \text{(S25)}$$
$$kon_{Gr}(t) = kon_{Gr}(0)\cdot\left(1+c\ PLD^*(t)\right) \qquad \text{(S26)}$$

The values for coefficients a, b and c are listed in the legends of Figs. 5d and S7 (a, b and c are 0 prior to stimulus (t < 60s) and jumped to the values listed on the respective legends for t >= 60 s). The functional form for Eqs.S23-26 and the coefficients a, b and c where chosen so that the phosphorylation of GDI would monotonically increase within about 10 minutes, and Rac would translocate within 20 minutes (11).

The concentration of PLD, its activation rates and the function PLD* were chosen in an arbitrary fashion, so that we could introduce the effect of granular fusion on change of lipid membrane

composition and affinity between Rac and the membrane. In the VCell model, the rates *pG*, kon_M and kon_{Gr} are P_G_s, konM_R1 and konGr_R1 respectively. The coefficients a, b and c from Eqs. S15-S17 are changePsGDI, changekonM and coef_on_Gr respectively. The variables on the plots of Figs. 5d and.S7: pS_GDI, cyt. Rac, gr. Rac, p.m. Rac and K_{D4} correspond to Group_gdiSp_c, Group_tot_Rac_C, Group_tot_Rac_G, Group_tot_Rac_M and KD4_sys_c respectively.

Figure S7a shows that by increasing the phosphorylation rate of GDI by up to five fold, results in up 2.1 fold increase in amount of phosphorylated GDI. The cytosolic Rac concentration drops by 20%, and membrane concentration of Rac increases up to 60%. Note that K_{Dm} is constant. Figure S7b shows that for a=1.5 only, the increase in phosphorylated GDI is between 60-78%, for b=1-8. For b=8, Rac cytosolic concentration dropped to 60% twenty minutes after the stimulus. But the concentration of Rac at the membrane increased beyond the experimental observations: by one order of magnitude rather than two fold. In contrast, the concentration of Rac at the granule membranes decreased by 35%. Notice that K_{D4} is no longer constant. By increasing the affinity between granule membranes and Rac in proportion to the affinity between Rac and the plasma membrane (Fig S7c), all experimental observations can be satisfied.

Figure S7

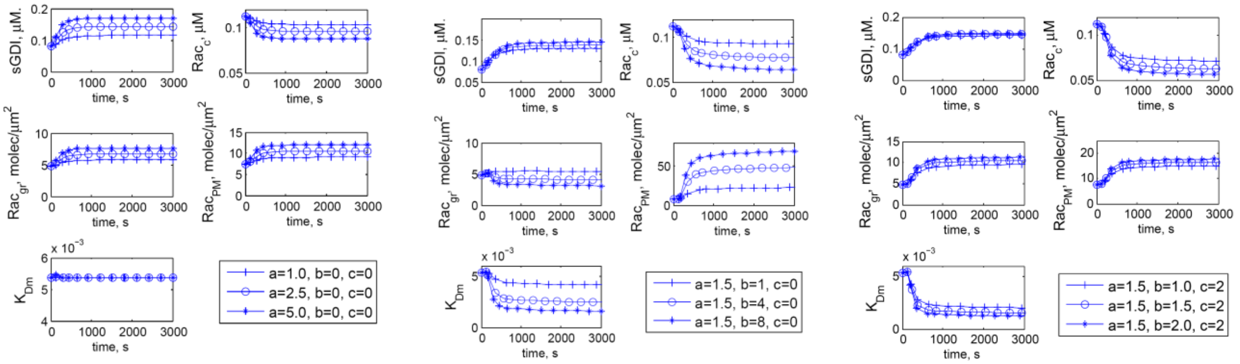

Figure S7. Transient translocation of Rac between cytosol, plasma and granular membranes upon GDI serine phosphorylation. The coefficients in the legends obey Eqs. S24-26. sGDI, concentration of serine phosphorylated GDI; Rac_c, cytosolic concentration of Rac; Rac_{gr}, concentration of Rac in granular membrane; Rac_{PM}, concentration of Rac in plasma membrane; K_{Dm}, ratio between free (not bound to GDI) cytosolic Rac and membrane (plasma plus granular) bound Rac.

Supporting References

1. White, F. M. 1988. Heat and mass transfer. Addison-Wesley, Reading, Mass.
2. Moissoglu, K., B. M. Slepchenko, N. Meller, A. F. Horwitz, and M. A. Schwartz. 2006. In vivo dynamics of Rac-membrane interactions. Mol Biol Cell 17:2770-2779.
3. Geiszt, M., M. C. Dagher, G. Molnár, A. Havasi, J. Faure, M. H. Paclet, F. Morel, and E. Ligeti. 2001. Characterization of membrane-localized and cytosolic Rac-GTPase-activating proteins in human neutrophil granulocytes: contribution to the regulation of NADPH oxidase. Biochem J 355:851-858.

4. Bielek, H., A. Anselmo, and C. Dermardirossian. 2009. Morphological and proliferative abnormalities in renal mesangial cells lacking RhoGDI. Cell Signal 21:1974-1983.
5. Blinov, M. L., J. R. Faeder, B. Goldstein, and W. S. Hlavacek. 2004. BioNetGen: software for rule-based modeling of signal transduction based on the interactions of molecular domains. Bioinformatics 20:3289-3291.
6. Faeder, J. R., M. L. Blinov, and W. S. Hlavacek. 2009. Rule-based modeling of biochemical systems with BioNetGen. Methods Mol Biol 500:113-167.
7. Matlab. Mathworks, Inc., Natick, MA.
8. Miura, Y., A. Kikuchi, T. Musha, S. Kuroda, H. Yaku, T. Sasaki, and Y. Takai. 1993. Regulation of morphology by rho p21 and its inhibitory GDP/GTP exchange protein (rho GDI) in Swiss 3T3 cells. J Biol Chem 268:510-515.
9. Jayaram, B., I. Syed, C. N. Kyathanahalli, C. J. Rhodes, and A. Kowluru. 2011. Arf nucleotide binding site opener [ARNO] promotes sequential activation of Arf6, Cdc42 and Rac1 and insulin secretion in INS 832/13 β-cells and rat islets. Biochem Pharmacol 81:1016-1027.
10. Li, J., R. Luo, A. Kowluru, and G. Li. 2004. Novel regulation by Rac1 of glucose- and forskolin-induced insulin secretion in INS-1 beta-cells. Am J Physiol Endocrinol Metab 286:E818-827.
11. Wang, Z., and D. C. Thurmond. 2010. Differential phosphorylation of RhoGDI mediates the distinct cycling of Cdc42 and Rac1 to regulate second-phase insulin secretion. J Biol Chem 285:6186-6197.
12. Ma, W. N., S. Y. Park, and J. S. Han. 2010. Role of phospholipase D1 in glucose-induced insulin secretion in pancreatic Beta cells. Exp Mol Med 42:456-464.
13. Jenkins, G. M., and M. A. Frohman. 2005. Phospholipase D: a lipid centric review. Cell Mol Life Sci 62:2305-2316.
14. Hughes, W. E., Z. Elgundi, P. Huang, M. A. Frohman, and T. J. Biden. 2004. Phospholipase D1 regulates secretagogue-stimulated insulin release in pancreatic beta-cells. J Biol Chem 279:27534-27541.
15. McDonald, P., R. Veluthakal, H. Kaur, and A. Kowluru. 2007. Biologically active lipids promote trafficking and membrane association of Rac1 in insulin-secreting INS 832/13 cells. Am J Physiol Cell Physiol 292:C1216-1220.
16. Wang, Z., E. Oh, and D. C. Thurmond. 2007. Glucose-stimulated Cdc42 signaling is essential for the second phase of insulin secretion. J Biol Chem 282:9536-9546.
17. Barg, S., L. Eliasson, E. Renström, and P. Rorsman. 2002. A subset of 50 secretory granules in close contact with L-type Ca2+ channels accounts for first-phase insulin secretion in mouse beta-cells. Diabetes 51 Suppl 1:S74-82.
18. Rorsman, P., and E. Renström. 2003. Insulin granule dynamics in pancreatic beta cells. Diabetologia 46:1029-1045.
19. Wang, Z., and D. C. Thurmond. 2009. Mechanisms of biphasic insulin-granule exocytosis - roles of the cytoskeleton, small GTPases and SNARE proteins. J Cell Sci 122:893-903.
20. MacDonald, P. E., and P. Rorsman. 2007. The ins and outs of secretion from pancreatic beta-cells: control of single-vesicle exo- and endocytosis. Physiology (Bethesda) 22:113-121.

```
## BNGL model for Rac cycling - C.V. Falkenberg, May, 2011
## OUTPUT files for ODE simulation (analogous file created for SSA simulation):
## simple.net         : species reaction network file.
## simple_ode.cdat    : ODE simulation state trajectory.
## simple_ode.gdat    : ODE simulation observables trajectory (units of molecules/simcell).
## simple_ode_end.net : network file set to end-of-simulation concentrations.
## simple.m  : matlab file

begin model
begin parameters

Sfc2Vol 0.524   # (1/um) # in vcell, remember to assign area and volume sizes.
Vol2Sfc      1/Sfc2Vol  #(um) # volume of compartment inside the membrane; or,membrane protein binding site face the volume

# in bng, units for membrane species are identical to cyt species; it's more convenient to use uM; for membrane properties, compute as
(number of moles in the membrane)/(volume of child compartment)
# but when exported, bng -> sbml -> vcell;
# (1) the values in sbml are assumed to be uMole/m2 for membrane concentrations;  (2) a second correction is necessary in order to
compute the membrane fluxes in vcell
toexport1      Vol2Sfc*1e-3   # in vcell, species at membrane are imported as if in uMole/m2  # converts uM into uMoles/m2 = sbml
default ; [uMoles/dm3=uMoles/(m2*mm)]*(1e-3*um/Sfc2Vol)
toexport2   1e-12  # in order to correct membrane fluxes converted from SBML to VCell

#-------  below, pick values for pmp and pmp0: -----
# now we collapse all corrections in 2 parameters to be used with IC and reaction rates:
# in oder to export the code:
#pmp        toexport1*toexport2   # parameter for membrane concentrations
#pmp0      toexport2                   # parameter for membrane rates

# if interested in using the code in bng or matlab:
pmp       1
pmp0     1
#------- ----------------- -----


## reaction rates to be used when runing bng

gef    18.79   # (1/s)   # optimized parameter
gap    10      # (1/s)


KD2G   1.3029e-4  # optimized parameter
KD2T   KD2G/0.9985  #optimized parameter
koffDG 1      # (1/s)
koffTG 1      # (1/s)
konDG  koffDG/KD2G   # bind rac_GDP to GDI    # (1/(uM s))
konTG  koffTG/KD2T   # bind rac_GTP to GDI    # (1/(uM s))


konE   0.1 # bind rac_GTP to effector  # (1/(uM s))
koffE  0.1    # (1/s)
```

```
konM    2.842   # Rac association with membrane   ( 1/s)      # optimized parameter
koffMD  0.1496  # complex racGDP dissociates from membr  # (1/s)# optimized parameter
koffMT  0.0111  # complex racGTP dissociates from membr  # (1/s)# optimized parameter



end parameters
begin molecule types    # define molecules present in the simulation


GDI(s1,loc~Cyt)
GDP(s2,loc~Cyt)
GTP(s2,loc~Cyt)

rac(s1,s2,loc~Memb)
G12V(s1,loc~Cyt)

eff(s1,loc~Memb)


end molecule types
begin seed species      # initial conditions


GDI(s1,loc~Cyt)       0.01194    # = 0.1385-0.12656  # uM      # optimized parameter: total GDI
rac(s1!1,s2!2,loc~Cyt).GDP(s2!2,loc~Cyt).GDI(s1!1,loc~Cyt)  0.12656   #  0.12656  = 0.0804+0.04616  when expressed wt; 0.0804 when
expressed G12V# uM   # optimized parameters: total endogenous Rac and transfected
rac(s1!1,s2!2,loc~Cyt).GTP(s2!2,loc~Cyt).GDI(s1!1,loc~Cyt)  0  # uM

G12V(s1,loc~Cyt)                             0  # 0  when expressed Rac wt, or   0.04616  when expressed G12V  #uM    # optimized
parameter: transfected G12V
G12V(s1,loc~Memb)                            0*pmp/pmp0 #uM

# all membrane concentrations must be converted when exported
rac(s1,s2!2,loc~Memb).GDP(s2!2,loc~Memb)                           0*pmp/pmp0     # uM or uMole/m2   # in vcell will be converted to
molec/um2
rac(s1,s2!2,loc~Memb).GTP(s2!2,loc~Memb)                           0*pmp/pmp0     # uM or uMole/m2   # in vcell will be converted to
molec/um2
eff(s1,loc~Memb)                                             0.5*pmp/pmp0   # uM or uMole/m2   # in vcell will be converted to
molec/um2


end seed species
begin observables       # model outputs

Molecules    rac       rac
Molecules   GDI        GDI

Molecules      tot_rac_M                    rac(loc~Memb)
Molecules      tot_rac_C                    rac(loc~Cyt)

Molecules g12V                G12V
```

```
Molecules g12V_c                      G12V(loc~Cyt)
Molecules g12V_m                      G12V(loc~Memb)

Molecules    rac_GDP_m        rac(s2!2,loc~Memb).GDP(s2!2,loc~Memb)
Molecules    rac_GTP_m        rac(s2!2,loc~Memb).GTP(s2!2,loc~Memb)
Molecules    rac_GTP_f        rac(s1,s2!2,loc~Memb).GTP(s2!2,loc~Memb)
Molecules    rac_GTP_mG               rac(s1!1,s2!2,loc~Memb).GTP(s2!2,loc~Memb).GDI(s1!1,loc~Memb)
Molecules    rac_GTP_E        rac(s1!1,s2!2,loc~Memb).GTP(s2!2,loc~Memb).eff(s1!1,loc~Memb)

Molecules    rac_GDP_c        rac(s2!2,loc~Cyt).GDP(s2!2,loc~Cyt)
Molecules    rac_GTP_c        rac(s2!2,loc~Cyt).GTP(s2!2,loc~Cyt)

Molecules    tot_GDP                  rac(s2!2,loc~Memb).GDP(s2!2,loc~Memb),GDP(loc~Cyt)
Molecules    tot_GTP                  rac(s2!2,loc~Memb).GTP(s2!2,loc~Memb),GTP(loc~Cyt)

Molecules      GDI_c   GDI(loc~Cyt)
Molecules      GDI_m   GDI(loc~Memb)

Molecules      GDI_mb  GDI(s1!1,loc~Memb)
Molecules      GDI_mf  GDI(s1,loc~Memb)
Molecules      GDI_cb  GDI(s1!1,loc~Cyt)
Molecules      GDI_cf  GDI(s1,loc~Cyt)


end observables

begin reaction rules

## all membrane reactions need to be re-scaled when exported by pmp0;
## the ones translocating  Cyt -> Memb (this direction only), or combining Memb+Memb, need to be corrected by pmp.

# gef, gap
rac(s1,s2!2,loc~Memb).GDP(s2!2,loc~Memb)                    <->     rac(s1,s2!2,loc~Memb).GTP(s2!2,loc~Memb)
       gef*pmp0, gap*pmp0
rac(s1,s2!2,loc~Cyt).GTP(s2!2,loc~Cyt)                              ->      rac(s1,s2!2,loc~Cyt).GDP(s2!2,loc~Cyt)
       gap*4
#binding to effector
rac(s1,s2!2,loc~Memb).GTP(s2!2,loc~Memb)+eff(s1,loc~Memb)          <->
       rac(s1!1,s2!2,loc~Memb).GTP(s2!2,loc~Memb).eff(s1!1,loc~Memb)              konE*pmp0*pmp0/pmp, koffE*pmp0
G12V(s1,loc~Memb)+eff(s1,loc~Memb)                          <->     G12V(s1!1,loc~Memb).eff(s1!1,loc~Memb)
       konE*pmp0*pmp0/pmp, koffE*pmp0

#translocation between m-c
rac(s1,s2!2,loc~Memb).GDP(s2!2,loc~Memb)                    <->           rac(s1,s2!2,loc~Cyt).GDP(s2!2,loc~Cyt)
       koffMD*pmp0,konM*pmp0
rac(s1,s2!2,loc~Memb).GTP(s2!2,loc~Memb)                    <->           rac(s1,s2!2,loc~Cyt).GTP(s2!2,loc~Cyt)
       koffMT*pmp0,konM*pmp0
G12V(s1,loc~Memb)                                           <->     G12V(s1,loc~Cyt)
       koffMT*pmp0,konM*pmp0

# binding to GDI
```

```
rac(s1,s2!2,loc~Cyt).GDP(s2!2,loc~Cyt)+GDI(s1,loc~Cyt)                     <->
       rac(s1!1,s2!2,loc~Cyt).GDP(s2!2,loc~Cyt).GDI(s1!1,loc~Cyt)          konDG, koffDG
rac(s1,s2!2,loc~Cyt).GTP(s2!2,loc~Cyt)+GDI(s1,loc~Cyt)                     <->
       rac(s1!1,s2!2,loc~Cyt).GTP(s2!2,loc~Cyt).GDI(s1!1,loc~Cyt)          konTG, koffTG
G12V(s1,loc~Cyt)+GDI(s1,loc~Cyt)                            <->    G12V(s1!1,loc~Cyt).GDI(s1!1,loc~Cyt)                        konTG,
koffTG



end reaction rules
end model



## model ACTIONS

# generate network of all species and reactions
#  with restrictions on iterations and complex size (aggregation)
generate_network({overwrite=>1,max_iter=>12,max_agg=>12});

writeSBML();                   # Output equations in SBML format.  # write before it runs so that ic are conserved
writeMfile();                   # Output equations as a Matlab m-file.

# Run an ODE simulation.  Results saved to files with prefix: "simple_ode"
simulate_ode({suffix=>ode,t_start=>0,t_end=>300,n_steps=>180});


# VCell commands:

#%VC% mergeReversibleReactions
#%VC% speciesRenamePattern("\." , "_")
#%VC% speciesRenamePattern("Cyt" , "")
#%VC% speciesRenamePattern("Memb" , "")
#%VC% speciesRenamePattern("Ext" , "")
#%VC% speciesRenamePattern("[\(,][a-zA-Z]\w*", "")
#%VC% speciesRenamePattern("~|!\d*", "")
#%VC% speciesRenamePattern("\(\)", "")
#%VC% speciesRenamePattern("\)", "")
#%VC% setUnit("all", "default")
#%VC% compartmentalizeSpecies("loc~Cyt", "3", "Cytoplasm","Membrane")
#%VC% compartmentalizeSpecies("loc~Memb", "2", "Membrane", "Extracellular")
#%VC% compartmentalizeSpecies("loc~Ext", "3", "Extracellular", "")
```
